# The predictive role of protease-activated receptor (PAR-1) polymorphisms and activated microplatelets on the severity of atherosclerosis – preliminary studies

**DOI:** 10.3389/fmolb.2025.1662954

**Published:** 2026-01-16

**Authors:** Urszula Jakobsche-Policht, Agnieszka Bronowicka-Szydełko, Rajmund Adamiec, Dorota Bednarska-Chabowska, Łukasz Lewandowski, Rafał Małecki, Kinga Gostomska-Pampuch, Joanna Adamiec-Mroczek, Marta Myszka-Kozłowska, Dagmara Baczyńska, Maciej Rabczyński, Edwin Kuźnik, Jacek Polański, Helena Martynowicz, Daria Dolińska, Paulina Matlak, Julia Sobczyńska, Maciej Ziomek, Maciej Tota, Wojciech Stach, Katarzyna Madziarska

**Affiliations:** 1 Department of Angiology and Internal Diseases, Institute of Internal Diseases, Wroclaw Medical University, Wroclaw, Poland; 2 Department of Biochemistry and Immunochemistry, Wroclaw Medical University, Wroclaw, Poland; 3 Clinical Department of Diabetology, Hypertension and Internal Diseases, Institute of Internal Diseases, Wroclaw Medical University, Wroclaw, Poland; 4 Department of Non-Procedural Clinical Sciences, Faculty of Medicine, Wroclaw University of Science and Technology, Wroclaw, Poland; 5 Clinical Department of Ophthalmology, Wroclaw Medical University, Wroclaw, Poland; 6 Department of Pediatric Bone Marrow Transplantation, Oncology and Haematology, Wroclaw Medical University, Wroclaw, Poland; 7 Department of Molecular and Cellular Biology Faculty of Pharmacy, Wroclaw Medical University, Wroclaw, Poland; 8 Faculty of Medicine, Wroclaw Medical University, Wroclaw Medical University, Wroclaw, Poland

**Keywords:** atherosclerosis obliterans, diabetic macroangiopathy, microplatelets, protease-activated receptor (PAR-1), plateletes

## Abstract

This study is a comprehensive analysis of PAR-1 – involved in thrombin interaction with platelets (PLT), present on PLT and microparticles (PMP) – to understand its role in diabetic macroangiopathy (DM) and atherosclerosis obliterans (AO). The applied RT-PCR, aggregometry, flow cytometry, a proprietary method for PMP level determination, ELISA, and multidimensional statistical analysis allowed for the determination of: PAR-1 activation levels, its polymorphisms, PLT/PMP aggregation capacity, hemostatic factors, and their interrelationships. In DM, the −506D/D and IVS-14A/A polymorphisms were significantly more frequent, whereas the −506I/D was much more common in AO, suggesting the protective properties of the I allele and its potential significance as a prognostic factor for a milder course of atherosclerosis. Similarly, increased PMP activity in DM indicates that activated PMP contribute to the atherosclerosis progression. A probable explanation for the reduced PAR-1 activation in AO is its association with the observed lower levels of von Willebrand factor. Interaction analysis showed that although the percentage of PMP did not affect the odds of AO (among AO and DM), at high PMP percentages, increased PAR-1 activation became a factor elevating the AO odds. Quantitative assessment of PAR-1 and PMP allows for predicting the severity of atherosclerosis.

## 1 Introduction

Atherosclerosis is a chronic and generalized inflammatory condition of the arteries, leading to degenerative and proliferative changes in the intima and media of the aorta, coronary arteries, cerebral arteries, and limb arteries (so-called macroangiopathies). The most significant organ complications of atherosclerosis include coronary artery disease, ischemic stroke, and chronic lower limb ischemia (Davies, 2012). It most commonly manifests as atherosclerosis obliterans (AO) or diabetic macroangiopathy (DM), which refers to widespread atherosclerotic changes in various arterial segments associated with type 2 diabetes (T2DM). Both of these conditions are considered major global health issues of the 21st century and are continuously increasing in prevalence. A meta-analysis has shown that peripheral artery disease (PAD) manifests as a chronic atherosclerotic process across all populations (Fowkes et al., 2013). The incidence of this disease is rising, particularly in low-income countries, highlighting that it is no longer confined to developed nations but has become a global concern. In 2010, the number of diagnosed atherosclerosis cases worldwide was approximately 202 million (Davies, 2012; Criqui and Aboyans, 2015). AO is a chronic, multifactorial disease affecting elastic and large muscular arteries, characterized by the accumulation of atherosclerotic plaques, which may undergo erosion or rupture, especially in high-risk cases known as “vulnerable plaques.” The underlying pathology involves a complex inflammatory process that damages blood vessel walls and promotes plaque formation due to both local and systemic factors (Jebari-Benslaiman et al., 2022). Inflammatory mechanisms play a key role not only in the onset of atherosclerosis but also in its progression and the rupture of plaques, leading to acute clinical complications (Ross R., 1999). AO is associated with arterial occlusions or stenoses, occurring from the suprarenal segment of the aorta to the arteries of the feet, ultimately leading to chronic lower limb ischemia. Atherosclerosis is the cause of 98% of lower limb ischemia cases (Conte et al., 2019; Jebari-Benslaiman et al., 2022). DM, on the other hand, is considered secondary atherosclerosis due to diabetes (Katakami, 2018), involving multiple molecular mechanisms that lead to dysfunction and damage of large blood vessels (Xiaoxu et al., n.d.). It frequently contributes to cerebrovascular disorders, ischemic heart disease, peripheral artery disease, and other vascular conditions, which significantly reduce the quality of life and even increase mortality among affected patients. Clinically, DM manifests as coronary artery disease, stroke, or chronic lower limb ischemia (Katakami, 2018). Atherosclerotic changes in diabetic patients develop earlier and progress more aggressively, with significantly worse prognosis. Compared to individuals with AO, those with DM have a 3- to 6-fold higher incidence and mortality rate from ischemic heart disease, and their risk of limb amputation is up to 13 times higher. Additionally, the treatment of diabetes and its vascular complications imposes significant financial burdens—in 2019, over 10% of total healthcare expenditures in Poland were allocated to diabetes-related care (Wang et al., 2022; Araszkiewicz et al., 2023). Understanding the complex mechanisms driving DM initiation and progression is crucial for developing effective diagnostic tools and therapeutic interventions (Xiaoxu et al., n.d.).

As atherosclerosis progresses, characteristic changes form in the vascular endothelium, beginning with primary hemostatic plugs, which later develop into atherosclerotic plaques. This process is degenerative but can undergo both progression and regression. While atherosclerosis was once considered an irreversible and inevitable degenerative process, current research highlights its dynamic nature, with increasing recognition of its variability. Clinical studies and data show that atherosclerotic plaques evolve in multiple directions—they may progress, regress, or even alter their properties, influencing the course of the disease. The primary cause of atherosclerosis is endothelial dysfunction, triggered by various factors involved in: lipid metabolism disorders, monocyte transmigration and their transformation into macrophages, immune responses, neovascularization, increased induction of blood coagulation processes, where platelets (PLT) play a crucial role. Inflammatory processes, leading to the formation of atherosclerotic plaques. Platelets play a key role in promoting atherosclerosis and serve as a link between hemostasis and inflammatory processes in the disease. These blood cells possess multiple adhesive glycoprotein receptors on their surface, allowing them to bind to various molecules: Glycoprotein GPIa binds to collagen, a major component of the subendothelial layer. Glycoprotein GPIb and the GPIIb/IIIa complex facilitate platelet binding to von Willebrand factor (vWF), which is released from platelets and endothelial cells and is essential for proper platelet adhesion to collagen (Zapata et al., 2014). The GPIIb/IIIa complex also functions as a receptor for fibrinogen, which bridges two platelets, playing a crucial role in platelet aggregation. These glycoproteins act as receptors for numerous factors that either activate or inhibit platelet function, highlighting their critical role in both coagulation and atherosclerotic inflammation.

On the surface of platelets, there are also thrombin-activated receptors known as PAR-1 (Protease-Activated Receptor-1), which are coupled with G proteins (G protein-coupled receptors, GPCR), forming the largest superfamily of cell surface receptors (Fredriksson et al., 2003). PAR-1 plays a crucial role in the thrombin signaling pathway (Rasmussen et al., 1991; Vu et al., 1991). PAR receptors bind to thrombin irreversibly, and signal transmission is terminated through the classical receptor desensitization mechanism. Thrombin-activated PAR-1 is expressed on the surface of various cell types, primarily those associated with the blood vessel wall (endothelial cells, fibroblasts, myocytes) (Austin et al., 2013), in all types of blood cells (platelets, neutrophils, macrophages), as well as in epithelial cells, neurons, astrocytes, immune system cells, and mast cells and macrophages present in the tumor microenvironment (Austin et al., 2013; Aisiku et al., 2015). Thrombin-activated PAR-1 induces the expression of various molecules on the surface of endothelial cells, including P-selectin and E-selectin, adhesion proteins ICAM-1 and VCAM-1, pro-inflammatory cytokines such as IL-6 and IL-8, platelet-activating factors (e.g., PAF), chemotactic proteins for monocytes, and the activation of cyclooxygenase-2 (COX-2). Chemotactic factors stimulate the migration of PLT and leukocytes to the site of their release. Interestingly, when PAR-1 is activated by activated protein C instead of thrombin, it exhibits cytoprotective properties (Ludeman et al., 2005). PAR-1 has a moderate affinity for thrombin (Kd ∼10 nM) and is present on the cell surface in quantities of approximately 1,500–2,000 copies (Vassallo et al., 1992).

So far, PAR-1 remains a poorly understood receptor. It is known to play a role in the activation of coagulation processes, dysregulation of hemostasis, and inflammation induction. It also contributes to platelet activation—specifically, activated PAR-1 leads to changes in platelet shape, causing the release of granules, platelet aggregation, and adhesion to the endothelium. PAR receptors facilitate thrombin interaction with PLT, which in turn triggers intracellular signaling pathways. This process results in the release of inflammatory factors, leukocyte recruitment, platelet adhesion to the endothelium (where von Willebrand factor, vWF, plays a key role), and the formation of edema. Activation of PAR receptors initiates intracellular signaling pathways that can proceed through various routes, depending on the G protein subtype involved. The variability in cellular responses to thrombin stimulation arises from this diversity in signaling mechanisms (Ossovskaya and Bunnett, 2004). Activated PAR receptors drive processes such as the release of inflammatory mediators, leukocyte recruitment, and edema formation. Four PAR receptor subtypes have been identified: PAR-1, PAR-2, PAR-3, and PAR-4, which share approximately 30% homology. Since PAR receptor activation is the first step in platelet-endothelium interaction, it is considered a key therapeutic and diagnostic target.

The aim of the study was to examine whether there are any genetic and molecular differences related to PAR-1 in patients with AO and MD. To achieve this, the expression of surface PAR-1 in PLT and platelet-derived microparticles (PMPs) activated by a synthetic thrombin analog (TRAP) in the context of atherosclerosis obliterans (AO), macroangiopathy in type 2 diabetes (DM), and a control group (without diagnosed atherosclerosis) was analyzed. Additionally, the study assessed the impact of selected genetic polymorphisms of PAR-1 on the development and prognosis of AO and DM. This allowed for an evaluation of whether mutations in the PAR-1 gene are associated with the prevalence of AO or DM. Another objective was to determine the relationship between PAR-1 concentration and selected anthropometric, metabolic, clinical, hemostatic, and inflammatory parameters. Furthermore, the study aimed to investigate the role of PMP activation in the pathogenesis of DM and AO and to assess the usefulness of flow cytometric imaging of PMPs.

## 2 Materials and methods

### 2.1 Determination of PAR-1 receptor activation level using flow cytometry

#### 2.1.1 Isolation and quantification of PLT

To determine the PLT count, a specific volume of PRP was transferred into a counting chamber containing a white bead. The sample was then diluted with an equal volume of procaine, mixed, and incubated for 10 min at room temperature. Next, a defined volume of the sample was applied to a Thoma counting chamber, incubated in a humidified chamber for 10 min, and the visible PLT were counted in five cross-sections of the grid. Quantitative assessment was performed using a Jenomed light microscope (Carl Zeiss, Germany). To obtain platelet-poor plasma (PPP), the PRP sample was further centrifuged (MPW-310, IKA, Poland) at room temperature for 15 min at 3,000 × g. The plasma was then carefully transferred to a plastic tube. A defined volume of the sample was subsequently applied to a Thoma counting chamber, incubated in a humidified chamber for 10 min, and the visible PLT were counted in five cross-sections of the grid. Quantitative assessment was performed using a Jenomed light microscope (Carl Zeiss, Germany).

#### 2.1.2 Platelet activation using thrombin receptor-activating peptide (TRAP)

500 μL of platelet-rich plasma was aliquoted into 200 μL portions, and 2 μL of eptifibatide at a concentration of 4 μg/mL was added to each sample. The obtained samples were incubated for 3 min to limit the formation of platelet aggregates. Next, 25 μL of thrombin receptor-activating peptide (TRAP) solution at 80 μM (Thrombin Receptor Activator Peptide 6, Sigma-Aldrich, Cat. No. T1573, St. Louis, MO, United States) was added to each sample and incubated in the dark for 10 min at room temperature. The activated material was then subjected to flow cytometric analysis.

#### 2.1.3 Labeling of PLT with PAR-1-APC antibodies

5 μL of the following samples were pipetted into plastic tubes: I) Isolated PLT II) Isolated PLT activated with TRAP. The samples were incubated for 40 min at room temperature in the dark. Next: For the PAR-1 test without PLT activation, 5 μL of PAR-1-APC antibodies at a concentration of 5 μg/5 × 10^5^ cells (Allophycocyanin (APC)-conjugated mouse monoclonal anti-human PAR-1; clone# 731115; mouse isotype: IgG1, R&D Systems, Minneapolis, Canada) and 5 μL of CD61-FITC antibodies (Monoclonal Mouse Anti-Human CD61, Platelet Glycoprotein IIIa/FITC, Clone Y2/51, code: F0803, DakoCytomation, Glostrup, Denmark) were added. For the PAR-1 test after PLT activation with TRAP, 5 μL of PAR-1-APC antibodies at a concentration of 5 μg/5 × 10^5^ cells and 5 μL of CD61-FITC antibodies were added. For the isotype control (negative control) after PLT activation with TRAP, 5 μL of ready-to-use IgG1 control antibodies (Negative Control Mouse IgG1, code: X0,931, DakoCytomation, Glostrup, Denmark) were added. For the platelet-selective marker test, 5 μL of CD61-FITC antibodies were added. The prepared samples were treated with a solution containing: 1) 1 mL of phosphate-buffered saline (PBS) pH 7.4 without Ca^2+^ and Mg^2+^ (Sigma-Aldrich, cat. no. T1573, St.Louis, MO, United States); 2) 0.5% bovine serum albumin (BSA) (Albumin Bovine Serum Minimum 98%, cat. no. A-7030-10G, Sigma-Aldrich, St. Louis, MO, United States); 3) 2 mM ethylenediaminetetraacetic acid, EDTA (Sigma-Aldrich, cat. no. T1573, St. Louis, MO, United States). The samples were then centrifuged **(**MPW-360 with a horizontal rotor, IKA, Poland) at room temperature for 5 min at 1,500 × g, and the supernatant was discarded. The resulting pellet of stained cells was resuspended in 1 mL of PBS without Ca^2+^ and Mg^2+^, pH 7.4**,** and transferred to tubes for Fluorescence Activated Cell Sorting (FACS), i.e., flow cytometry. The reading and analysis of the samples were performed using a flow cytometer (BD FACSCanto™ Clinical Flow Cytometry System, BD Biosciences, San Jose, Canada).

#### 2.1.4 Determination of PAR-1 expression at the transcriptional level

To determine the expression levels of the PAR-1 gene and the β-actin control gene, mRNA isolated from human lymphocytes (stored at −80 °C) was subjected to reverse transcription polymerase chain reaction (RT-PCR). The experiment was conducted according to the manufacturer’s protocol for the TaqMan® Reverse Transcription Reagents (cat. no. N808234, Applera, Warsaw, Poland). Each reaction mixture (25 μL) contained: 1) template RNA: mRNA isolated from patient blood samples (1–3 μL, depending on RNA concentration); 2) dNTP mix (dATP, dGTP, dCTP, dTTP): 0.5 µM (5 μL); 3) magnesium chloride (MgCl_2_): 5.5 mM (5 μL); 4) reverse transcriptase enzyme: 50 U (0.63 μL); 5) PCR buffer: 2.5 μL; 6) random hexamer primers: 2.5 μM (1.25 μL); 7) RNase inhibitor: 20 U/L (0.5 μL); 8) RNase-free water (H_2_O RNase-free): 11.5–13.5 μL. The RT reaction was performed in 30 cycles with the following conditions: Incubation: 10 min at 25 °C; prolongation: 30 min at 48 °C; deactivation: 5 min at 95 °C. In parallel, RT-PCR was conducted for the reference genes β-actin and 14-3-3 under identical conditions. The obtained cDNA was then subjected to quantitative PCR (qPCR) using the TaqMan® Fast Universal PCR Master Mix, No AmpErase®UNG (cat. no. 4352042, Thermo Fisher Scientific, Massachusetts, GA, United States), following the manufacturer’s protocol. The results were analyzed using a real-time PCR system, and relative gene expression levels were calculated using the ΔΔCt method, with β-actin as the housekeeping gene for normalization (TaqMan®Fast Universal PCR Master Mix, No AmpErase®UNG nr. cat. 4352042, Thermo Fisher Scientific, Massachusetts, GA, United States).

### 2.2 Study of polymorphisms within the PAR-1 gene

#### 2.2.1 DNA isolation from whole blood

To determine genetic polymorphisms within the PAR-1 gene [i.e., 506 I/D (dbSNP: rs35900074), 1426 C/T (dbSNP: rs32934), IVSn-14 (dbSNP: rs168753)] in the study and control groups, it was necessary to isolate DNA from blood samples of all patients. These samples (previously collected in EDTA tubes (BD, Vacutainer K3E 7.2 mg, Wokingham, United Kingdom) in a volume of 100 μL and frozen at −80 °C) were processed according to the manufacturer’s protocol (Blood Mini, cat. no. 022-50, A&A BIOTECHNOLOGY, Gdańsk, Poland). After the experiment, quantitative and qualitative assessments of DNA were performed spectrophotometrically for each sample using a UV-VIS spectrophotometer (Semco S91E, Warsaw, Poland) based on the following principles.

#### 2.2.2 Quantitative assessment

$$1A_{260}\text{ Unit of dsDNA}=50\mu g/\text{mL }H_{2}O$$

$$1A_{260}\text{ Unit of ssDNA}=33\mu g/\text{mL }H_{2}O$$

The DNA concentration in the tested samples (volume = 50 μL ±2 μL) ranged from 350 to 980 μg/mL.

#### 2.2.3 Qualitative assessment

The purity of the DNA samples was evaluated by calculating the A260/A280 ratio (expected value: 1.8–2.05). All analyzed samples had values within the range of 1.8–2.0.

#### 2.2.4 Primer synthesis for PCR

The synthesis of forward/reverse primer pairs for the PAR-1 gene polymorphisms, i.e., −506 I/D (a mixture of alleles I and D), −1426 C/T, IVSn-14 A/T, as well as the reference gene β-actin (with a constant, known expression level, allowing PCR process control), was conducted at the DNA Sequencing Laboratory, IBB PAN; Oligo.PL. For the −506 I/D and −1426 C/T polymorphisms, only classical PCR (using a single primer pair) was planned. However, for the IVSn-14 A/T polymorphism, due to the specificity of the gene’s DNA sequence (i.e., the proximity of the transcription start site to the target transversion location), a two-step PCR was necessary using two pairs of primers. In the first PCR reaction, a longer fragment was amplified, while in the second, the obtained product (which was additionally purified before the next PCR) was used.

#### 2.2.5 Polymorphism 506 I/D–Insertion at position 506 (13 bp, two alleles: I and D)

To confirm the insertion at position −506 (13 bp) in the PAR-1 gene, a specific primer pair flanking the polymorphic site was used for gene amplification. The reaction mixture (25 μL) included: 0.2 μM forward primer (1 μL); 0.2 μM reverse primer (1 μL); 1.5 μL DNA template (isolated from the patient’s blood sample); 0.2 mM dNTP mix (1 μL) (Sigma-Aldrich, T1573, St. Louis, MO, United States); 1.25 mM MgCl_2_ (1 μL); 0.5U Taq polymerase (0.5 μL) (BioLabs, MA, United Kingdom); PCR buffer (2.5 μL); H_2_O_MilliQ_ (16.5 μL); PCR conditions (30 cycles): Initial denaturation: 94 °C, 3 min; Denaturation: 94 °C, 30 s; Annealing: 56 °C, 30 s; Polymerization: 72 °C, 1.15 min; Extension: 72 °C, 2 min; Final storage at 4 °C. The reference β-actin gene PCR was performed under the same conditions. PCR products were analyzed using electrophoresis on a 3% agarose gel containing ethidium bromide (0.5 μg/mL) at 80V for 1 h and 20 min at room temperature. UV detection was performed (Gel Logic 100 system, Kodak, NY, United States). Expected product sizes: 103 bp and 90 bp (I/D), 169 bp and 123 bp (II), 169 bp and 110 bp (DD), 568 bp (β-actin).

**Polymorphism 1426 C/T**–*Transition C → T, nucleotide 1426, upstream of the translation start site, in the regulatory region–the change creates a restriction site for the MvaI enzyme–the enzyme digests the amplified sequence into two fragments*: *391 bp and 39 bp, whereas the wild-type allele remains undigested*

The analysis of the −1426 C/T polymorphism was conducted using the PCR-RFLP method. A fragment of the PAR-1 gene, where a potential SNP polymorphism could be present, was amplified using forward and reverse primers, whose sequences are shown. The reaction mixture (25 μL) was prepared by combining: 0.5 μL of polymerase (BIOTOOLS B&M Labs, S.A. 10.036, Madrid, Spain), 50 mM MgCl_2_ (1 μL), dNTPs Mix 5U/μL (1 μL), PCR reaction buffer (2.5 μL), H_2_O_miliQ_ (16.5 μL), 1.5 μL of the template obtained after prior digestion of 4 μL of isolated DNA (from patient blood) with the MvaI restriction enzyme (1U, Fermentas, no. cat. ER0551, Multiscan FC Thermo Fisher Scientific, Massachusetts, GA, United States) in a volume of 2 μL Tango buffer and 12 μL H_2_O_miliQ_ at 37 °C for 2 h. The PCR reaction was performed for 30 cycles under the following time and temperature conditions: Initial denaturation (94 °C, 3 min), denaturation (94 °C, 30 s), annealing of primers to the template (56 °C, 30 s), polymerization (72 °C, 1.15 min), final extension (72 °C, 2 min). After the completion of the PCR reaction, samples were stored at 4 °C. In parallel, under the same time and temperature conditions, a PCR reaction was conducted for the reference β-actin gene. The PCR product was separated by electrophoresis on a 3% agarose gel containing ethidium bromide (0.5 μg/mL). Electrophoresis was conducted at 75 V for 1 h at room temperature. Detection of the products was performed under UV light (Gel Logic 100 system, Kodak, NY, United States). The expected product sizes were as follows: 430 bp (CC), 430 bp and 391 bp (CT), 391 bp and 39 bp (TT), 567 bp (β-actin).

**Polymorphism IVSn-14 A/T**–*A → T transversion in the intervening sequence (IVS), located 14 nucleotides upstream of the exon 2 start site*

The IVSn-14 A/T polymorphism in the PAR-1 gene was analyzed using the SNaPshot technique (single-nucleotide extension reaction). In the first amplification step, a longer fragment of the gene was amplified and hybridized with a pair of primers, IVSF and IVSR. The reaction mixture (25 μL) contained: 0.2 μM forward primer (1 μL), 0.2 μM reverse primer (1 μL), 1.5 μL of the template (DNA isolated from a patient blood sample), 0.2 mM dNTP mix: dATP, dGTP, dCTP, and dTTP (1 μL) (Sigma-Aldrich, T1573, St. Louis, MO, United States), 1.25 mM MgCl_2_ (1 μL), 0.5U Taq polymerase (0.3 μL) (BioLabs, MA, United Kingdom), PCR reaction buffer (2.5 μL), H_2_O_miliQ_ (16.7 μL). The initial PCR reaction was performed in 35 cycles under the following conditions: denaturation (96 °C, 10 s), annealing of primers to the template (53 °C, 30 s), polymerization (60 °C, 30 s), pause (4 °C, 2 min). The composition of the obtained PCR reaction mixture was verified using horizontal electrophoresis in a 1% agarose gel under the following conditions: 75 V, 1 h, at room temperature. The next step involved removing single nucleotides and excess primers from the reaction mixture (4 μL) using a 1.1 μL mixture of restriction enzymes, including: alkaline phosphatase (1 μL) to eliminate unincorporated dNTPs (Shrimp Alkaline Phosphatase, SAP, Fermentas, Vilnius, Lithuania), exonuclease (0.1 μL) to remove excess primers (ExoI, Exonuclease I, Fermentas, Vilnius, Lithuania). The digestion reaction was carried out for 30 min at 37 °C, followed by enzyme inactivation through 15-min denaturation at 80 °C. The samples were stored at 4 °C. The next step involved performing another PCR reaction using a second pair of primers, IVSF/SNP and IVSR/SNP, complementary to the sequence adjacent to the SNP site, in a minisequencing reaction using the SNaPshot Multiplex Kit (Applied Biosystems, Thermo Fisher Scientific, Massachusetts, GA, United States). The reaction mixture (5 μL) contained: 1 μL of the digested PCR product, 0.1 μM forward and reverse primers (1 μL), reaction mix (1.5 μL), H_2_O_miliQ_ (2.5 μL). This PCR reaction was performed in 25 cycles under the following conditions: denaturation: 96 °C, 10 s, primer hybridization: 57 °C, 30 s, elongation: 60 °C, 30 s. After PCR completion, unreacted dideoxynucleotides (ddNTPs) were digested using 1U SAP through incubation at 37 °C for 30 min, followed by enzyme inactivation *via* 15-min denaturation at 80 °C. For capillary electrophoresis, samples were prepared by adding 1 μL of the PCR SNaPshot product (digested with restriction enzymes) to 10 μL of formamide mixture, containing the LIZ 120 size standard (Applied Biosystems, no. cat. 4324287, Thermo Fisher Scientific, Massachusetts, GA, United States), and denaturing at 95 °C for 5 min. Separation and detection of products were performed using capillary electrophoresis in a genetic analyzer (ABI PRISM 3130 Genetic Analyzer, Applied Biosystems, Thermo Fisher Scientific, Massachusetts, GA, United States). The results were analyzed using an internal size standard (GeneScan 120LIZ-Size Standard 800Loads, Applera Polska, no. cat. 4324287, Warsaw, Poland) with GeneMapperID v3.2 software (Applied Biosystems, Thermo Fisher Scientific, Massachusetts, GA, United States).

### 2.3 Statistical analysis of results

The descriptive statistics used in the study were: mean, standard deviation, median, maximum, and minimum. A significance level of α = 0.05 was assumed for all tests. The Shapiro-Wilk test was used to verify the normality of the distribution of features. To assess differences in the distribution of features between the analyzed groups, the Wilcoxon rank-sum test and Welch’s t-test for unpaired variables were applied. The independence of categorical variables was tested using the Chi-square test. Moreover, differences between DM and AO were examined using modeling the odds of atherosclerosis occurrence among study participants diagnosed with one of the mentioned diseases. Logistic regression was employed for this purpose: univariate, multivariate based on effects (variables), and multivariate based on effects and interactions (second degree) between them. For this analysis, the Statistica 13.3 program, licensed by the Medical University of Silesian Piasts in Wroclaw, was used. Prior to the analysis, data cleaning was conducted by removing extreme values (by excluding the record from the database intended for further analysis). Additionally, the assumption of linearity with respect to the logarithm of the analyzed odds was checked (Box-Tidwell test). All quantitative variables were centered on the median to better fit the logit function to the data. The construction of the multivariate model based on effects was based on selecting the most multivariately significant variables through iteration (backward stepwise method, inclusion/exclusion criterion: p = 0.05). The decision to include variables in the model was based on the Wald statistic. For removing variables from the model, the Lagrange statistic (so-called “Score test”) was used. Multivariate models showing selected second-degree interactions were created through an initial evaluation of the significance of all possible interactions relative to the naïve model (LR type 1 test), followed by selecting those interactions that remained significant in the factor model (i.e., if the effects are A and B, the model includes: A, B, and the interaction A*B). Data visualizations were created in Python 3.10.7 (packages: pandas 1.4.4, numpy 1.21.4, matplotlib 3.5.3, seaborn 0.11.2).

## 3 Results

The study group consisted of blood/plasma sera taken from 95 patients at the Clinical Department of Angiology, Hypertension, and Diabetology, University Hospital in Wroclaw: 34 patients with diabetic macroangiopathy (DM) in the course of T2DM (mean age 60.88 ± 7.51 years), 43 patients with atherosclerosis obliterans, AO (mean age 56.69 ± 5.72 years), and 18 individuals without diabetes or atherosclerosis–the control group (mean age 57.50 ± 2.12 years). The youngest possible group of patients with peripheral artery disease (PAD) in stage IIB according to Fontaine (ABI <0.5) was selected for the study. Patients with T2DM were undergoing insulin therapy in combination with oral hypoglycemic medications. All participants were excluded from the study if they had an acute or chronic inflammatory condition, particularly tissue necrosis or a diagnosed cancer. All patients gave informed consent to participate in the study, which was approved by the Bioethics Committee of the Medical University of Wroclaw (No. KB – 174/2011, KB – 125/2024). A general characterization of the studied groups: assessment of anthropometric, laboratory, and clinical features, quantitative assessment of selected hemostasis and inflammation parameters of AO and DM are described in the Supplementary Material. Moreover Evaluation of PMPs and PLT aggregation is also presented in the Supplementary Material.

### 3.1 Assessment of surface PAR-1 activation level

**FIGURE 1:**
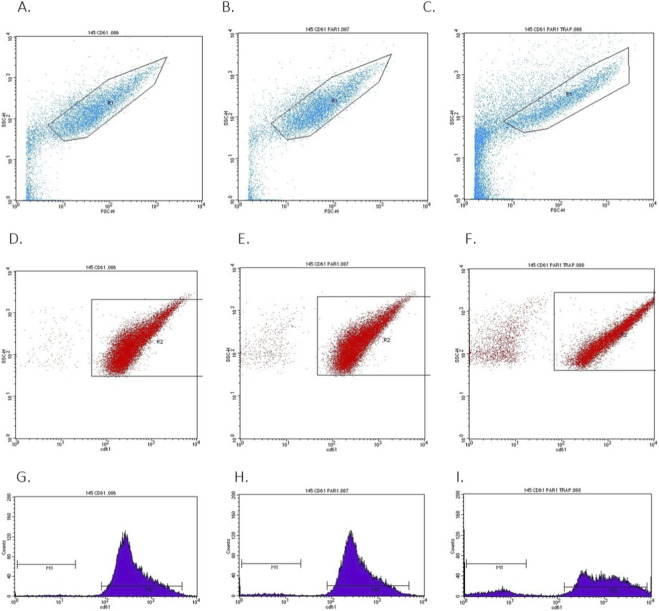
The cytometric analysis (SSC and FCS) of isolated human resting platelets **(A, D)**, gated and labeled PAR-1 without activation **(B, E)**, and gated and labeled PAR-1 with activation by 10 µM TRAP **(C, F)**; labeled with anti-CD61-FITC and PAR-1-APC antibodies **(G–I)**. The level of PAR-1 expression was read from gates P1. Markers M1 and M2 indicate the gates for microparticles and normal platelets, with the PAR-1 analysis applied to the summed population. An example image from a patient with DM is shown.

To determine the level of PAR-1 activation by TRAP, a laboratory test was performed to measure the PAR-1 concentration using PAR-1-APC labeled antibodies in plasma samples, both with activated and non-activated PLT. Control samples were simultaneously tested to detect any potential cross-reactivity that might occur between the components of biological material samples and PAR-1-APC antibodies. The evaluation of activated and non-activated PAR-1 levels was conducted using flow cytometry (Figure 1).

The cytometric analysis involved the initial identification and localization of platelets according to FSC (Forward Scatter), differentiating structures based on size, and SSC (Side Scatter), differentiating structures based on the density of internal granules. For this purpose, the R2 gate was set in the statistical analysis of the FACS Canto BD cytometer program; the number of recorded events in the flow of 10,000 cell objects was automatically counted. Next, the level of PAR-1 fluorescence labeled with APC on the platelet surface, labeled with CD61-FITC, was analyzed; the P1 gate was set for this purpose. To determine the correct/desired platelet population, markers M1 and M2 were analyzed for the studied structures. Data analysis was performed for individual patients in two experimental models: in isolated platelets before and after TRAP activation. The presented images suggest a significant increase in the number of PAR-1 receptors labeled on the surface of platelets after TRAP stimulation. The observed increase in the PAR-1 receptor activation level was particularly visible in patients with DM.

**FIGURE 2:**
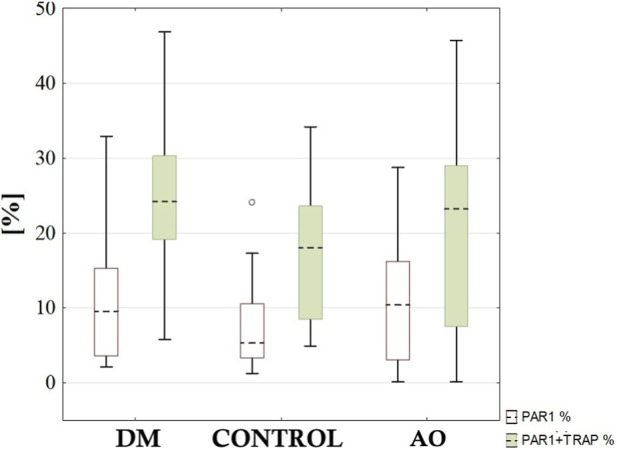
The percentage of PAR-1 receptor expression before and after the addition of the thrombin receptor activating peptide (TRAP) in blood samples from patients with diabetic macroangiopathy (DM), the control group (CONTROL), and atherosclerosis obliterans (AO).

A comparative analysis was conducted on the results showing the PAR-1 expression level before and after TRAP addition in plasma samples from patients with AO, DM, and the control group. It was known that the plasma from these patients did not differ significantly in terms of platelet count (248.59 ± 66.72; 271.56 ± 74.02; 252.94 ± 97.92, respectively) and mean platelet volume (MPV) (10.56 ± 1.24; 10.10 ± 1.15; 9.63 ± 1.09, respectively). The comparative analysis describing the level of PAR-1 expression on activated and non-activated platelets using TRAP in the plasma of patients with DM, AO, and the control group is shown in Figure 2.

The statistical analysis showed the following: The level of PAR-1 expression without TRAP activation did not significantly differ between the plasma of patients in the DM, AO, and control groups. A significant increase in the level of PAR-1 expression upon TRAP activation was observed in the DM group compared to the AO and control groups (25.31% ± 10.18% vs. 18.63% ± 12.98% vs. 17.14% ± 8.72%; p = 0.030, p = 0.011, respectively). These results indicate the significant role of the patomechanism involving PAR-1 and thrombin in the initiation and progression of atherosclerosis in the context of DM. A similar analysis was conducted in the same patient groups (DM, AO and control), considering gender. The statistical analysis revealed: In the female group, no significant difference was observed in the level of PAR-1 expression both before (8.85% ± 8.96% vs. 4.61% ± 4.41% vs. 7.74% ± 6.30%; p = 0.100, p = 0.682, respectively) and after PLT activation with TRAP (21.69% ± 10.08% vs. 21.85% ± 11.96% vs. 15.54% ± 8.56%; p = 0.925, p = 0.157, respectively). Among men, a significant increase in the level of PAR-1 without TRAP activation was found in the DM group compared to the control group (12.42% ± 8.82% vs. 5.27% ± 4.01%; p = 0.043) and in the AO group compared to the control group (11.56% ± 7.12% vs. 5.27% ± 4.01%; p = 0.046). Additionally, a significant increase in PAR-1 expression after TRAP activation was found in the DM group compared to the AO group in men (27.48% ± 10.03% vs. 18.18% ± 13.16%; p = 0.046). Furthermore, a comparative analysis was conducted on PAR-1 expression levels before and after TRAP activation in the various disease groups (DM, AO and control), considering gender. The statistical analysis showed that the level of PAR-1 expression before TRAP activation in the female AO group was significantly lower than in the male DM group (4.11% ± 4.18% vs. 11.85% ± 7.05%; p < 0.001) and all patients in the AO group (4.11% ± 4.18% vs. 9.69% ± 7.24%; p = 0.008). In the remaining groups, no statistically significant changes in PAR-1 expression levels were observed (both before and after activation with TRAP), although in the DM female group, a slight decrease in PAR-1 expression was noted compared to both the male DM group and all patients in the AO group; this relationship was not observed in the control group.

### 3.2 Determination of PAR-1 expression at the transcriptional level

PAR-1 expression can be regulated at the transcriptional level. To quantitatively assess gene expression at the mRNA level, real-time RT-PCR was performed. The material from 85 patients was analyzed–only from this number of samples was it possible to obtain a sufficient amount of material to make a reliable quantitative assessment of PAR-1 (ΔCT value below 30). The results are presented in Table 1.

**TABLE 1:** Gene expression level of PAR-1 and reference gene β-actin [ΔCT] in whole blood.

Studied parameters	Patients with DM (n = 30)			Patients with AO (n = 38)			Control group (n = 17)			p = 1 v 2	p = 1 v 3	p = 2 v 3
	1			2			3					
PAR1 [ΔC_T_]	1.12	±	0.41	1.72	±	1.67	0.67	±	0.03	**0.667**	**0.888**	**0.479**
β-actin [ΔC_T_]	0.07	±	0.13	0.06	±	0.07	0.07	±	0.05	**0.993**	**0.999**	**0.992**

The PAR-1 values ranged from 0.03 to 1.72. However, no statistically significant differences were observed. The obtained values did not correlate with either the number of PAR-1, receptors or with polymorphisms.

### 3.3 Determination of PAR-1 gene polymorphisms

**FIGURE 3:**
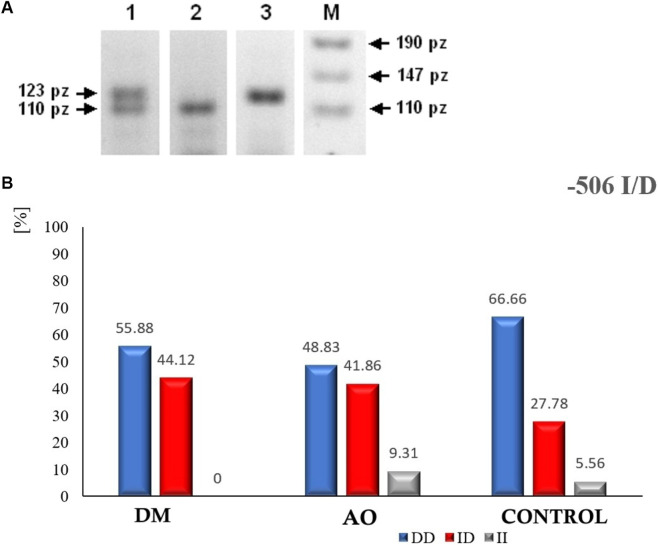
**(A)** Separation of DNA molecules in a 3% agarose gel of PAR-1 gene amplification products with the −506 I/D polymorphism. Lanes: 1 – homozygous I/I, 2 – heterozygous I/D, 3 – homozygous D/D, M–GeneRuler™ 50bp DNA Ladder (Fermentas). **(B)** The percentage distribution of the −506 I/D polymorphism variants in the PAR-1 gene: homozygous D/D (blue), heterozygous I/D (red), and homozygous I/I (green).

The predisposition of the platelet receptor PAR-1 to increased expression due to activation by a thrombin substitute, such as TRAP, could result from mutations that may appear in the gene encoding PAR-1, e.g., 506 I/D (insertion at position 506, 13 bp, involving two alleles: I and D), 1426 C/T (transition C → T), IVSn-14 (transversion A → T in the IVS intermediate sequence). To check for the presence of mutations in the PAR-1 encoding gene, PCR reactions were performed on DNA molecules (previously purified and isolated from patient plasma samples) containing the appropriate fragment of the PAR-1 gene (as a so-called template). PCR reactions were carried out under conditions suitable for amplification of the specific PAR-1 gene fragment (i.e., covering the potential mutation site characterizing the given polymorphism). The obtained products were subjected to either agarose gel electrophoresis (506 I/D, 1426 C/T) or capillary electrophoresis in a genetic analyzer (IVSn-14) to confirm or exclude the presence of a specific mutation—in other words, to determine the variant of the gene polymorphism. A photo of the three possible variants of the 506 I/D polymorphism of the PAR-1 gene is shown in Figure 3A.

The analysis of the presented gels confirmed the presence of three variants of the 506 I/D polymorphism in the PAR-1 gene in the material obtained from the patients involved in this study: homozygous I/I (lane 1), heterozygous I/D (lane 2), and homozygous D/D (lane 3). The percentage distribution of each polymorphism in the groups of patients with DM, AO, and the control group is shown in Figure 3B.

Based on the obtained results, it was found that the highest percentage of the −506 I/D heterozygote in relation to the −506 D/D homozygote was observed in the group with AO. The II variant occurred infrequently in each of the studied groups (<10.0%). In each group, the −506 D/D homozygotes were dominant.

**FIGURE 4:**
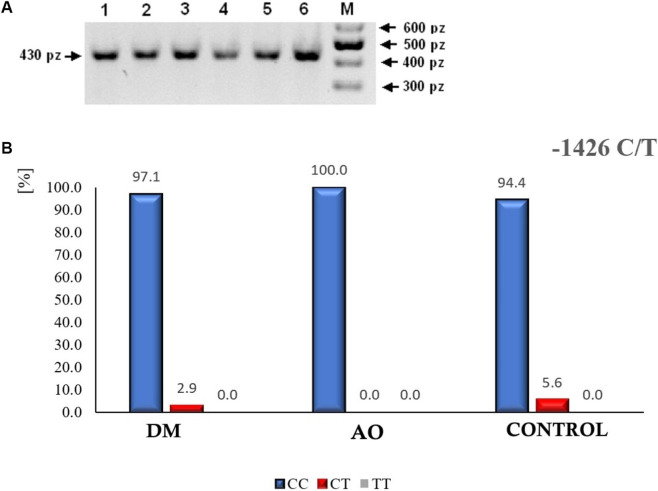
**(A)** The result of the restriction digestion of PCR products with the MvaI enzyme to check for the presence of the −1426 C/T polymorphism in the PAR-1 gene. Lanes: 1 – 6 homozygotes C/C, M–GeneRuler™ 100bp DNA Ladder (Fermentas). **(B)** The percentage distribution of the variants of the −1426 C/T polymorphism in the PAR-1 gene: homozygote C/C (blue color), heterozygote C/T (red color), homozygote T/T (green color).

In the designed experiment, aimed at checking the presence of possible variants of the −1426 C/T polymorphism in the PAR-1 gene (isolated from material obtained from patients included in this study), the molecular feature of the PAR-1 fragment resulting from the −1426 C/T mutation was utilized. The cytosine-to-thymine change at position −1,426 generates a restriction site for the MvaI enzyme. The mutated sequence can thus be cleaved by this enzyme into two fragments (391 bp and 39 bp in length). The wild-type allele, on the other hand, is not restricted (430 bp). Example products obtained from DNA amplification, which were subsequently treated with MvaI, are shown in Figure 4.

The analysis of six randomly selected products obtained from the PCR reaction and restriction digestion with the MvaI enzyme of DNA templates isolated and purified from biological material of patients involved in this study showed the presence of only the C/C homozygote–no −1426 C/T polymorphism was detected in any of the samples. The percentage distribution of the different variants of the −1426 C/T polymorphism in the PAR-1 gene is shown in Figure 4B.

**FIGURE 5:**
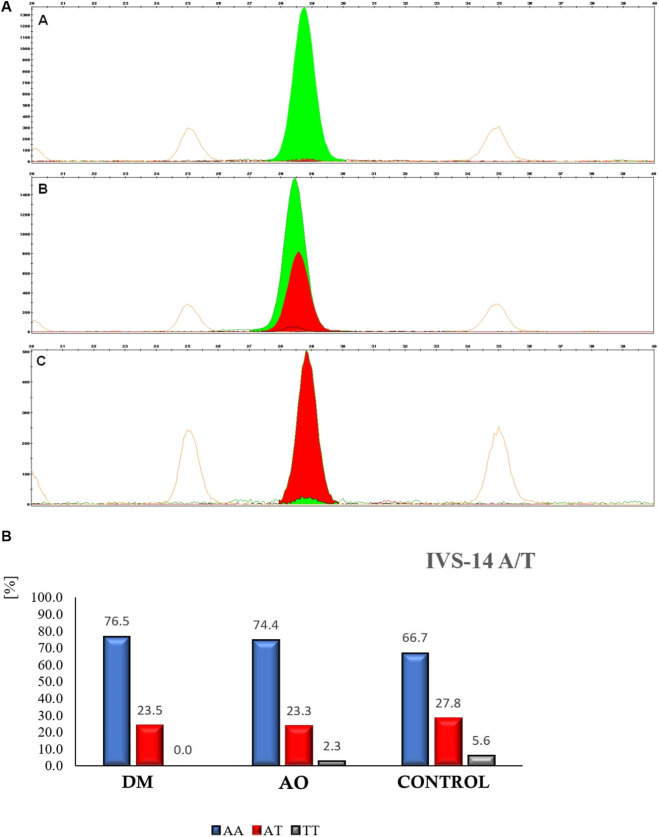
**(A)** Example separations of amplification products of the DNA fragment encompassing the IVSn-14 A/T polymorphism site of the PAR-1 gene using the SNaPshot method. Alleles were determined based on the size of primers and the colors of fluorescently labeled ddNTPs (terminators) incorporated during the primer extension reaction. **(A)** red peak, wild-type homozygote (AA); **(B)** green and red peaks, heterozygote (AT); **(C)** green peak, mutated homozygote (TT). **(B)** The percentage distribution of the variants of the IVS-14 A/T polymorphism of the PAR-1 gene is as follows: homozygote A/A (blue color), heterozygote A/T (red color), and homozygote T/T (green color).

Analysis of PCR products from the PAR-1 gene with the potential −1426 C/T mutation (obtained from biological material samples of all patients involved in this experiment) confirmed that in each of the studied groups, the highest percentage was homozygotes CC, heterozygotes CT occurred very rarely, and homozygotes TT were not present at all. Genotyping of the IVSn-14 A/T polymorphism in the PAR-1 gene was performed using the SNaPshot method, which involves the separation and detection of products by capillary electrophoresis relative to an internal size standard. This method revealed that the possible variants of this polymorphism were: homozygote wild type (AA), B–heterozygote (AT), C–homozygote mutated (TT). Example variants of the separation of IVSn-14 A/T polymorphism products of the PAR-1 gene are shown in Figure 5A.

The analysis of the results obtained using the SNaPshot method revealed the possibility of three variants of the IVSn-14 A/T polymorphism of the PAR-1 gene, namely, the wild-type homozygote (AA), heterozygote (AT), and mutated homozygote (TT). The percentage distribution of the various variants of the IVS-14 A/T polymorphism of the PAR-1 gene is shown in Figure 5B.

The analysis of the results showed that the percentage of homozygote AA was the highest in each of the studied groups, the heterozygote AT occurred less frequently and with comparable frequency across the groups, while homozygote TT was very rare in the IVS-14 A/T polymorphism of the PAR-1 gene. It was shown that the allele frequencies for 1426 T, 506 I, and IVSn-14 T were 0.028, 0.27, and 0.194, respectively. These were similar to the allele frequencies obtained in a study with a significantly larger group of patients (n = 1,214), where they were 0.041, 0.256, and 0.185, respectively. No significant deviation from Hardy-Weinberg equilibrium was observed for the studied polymorphisms in the studied population. No statistically significant difference was found in the occurrence of the −1426 C/T genotypes between the AO group and the control group, as well as between the DM group and the control group. A statistically significant difference was found between the occurrence of the IVS-14 A/T genotype (p = 0.015) in the AO and DM groups compared to the control group due to the increased frequency of the AA genotype (p = 0.001). A significant difference in the frequency of the −506 I/D genotype (p = 0.001) was observed between patients with DM and the control group, and between patients with AO.

**FIGURE 6:**
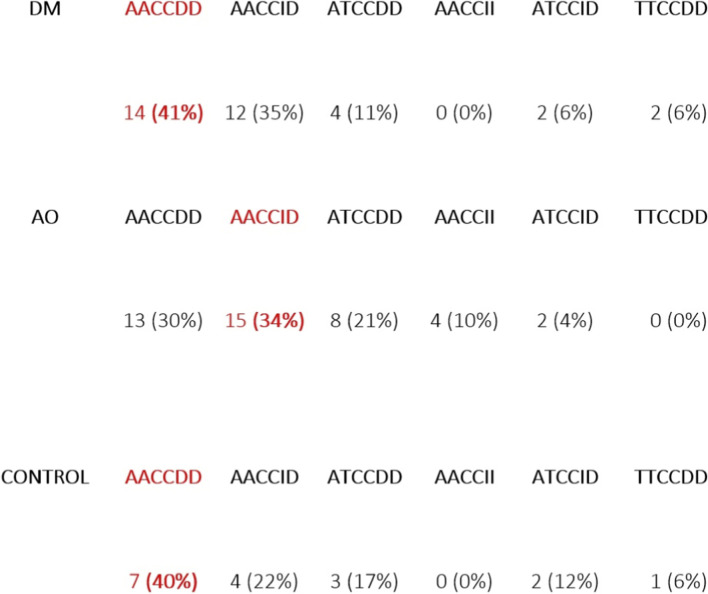
Comparison of study results in DM, AO, and the control group.

Additionally, an analysis was performed to determine the dominant genotypes in the DM, AO, and control groups. A summary of the percentage distribution of genotypes (−506, −1,426, IVSn-14) in the studied conditions and in the control group is presented in Figure 6.

Based on the conducted studies, it was found that within the three polymorphisms of the PAR-1 gene in DM, the dominant genotype is AACCDD (41%), similar to the control group (40%), while in AO, the dominant genotype is AACCID (34%).

### 3.4 Analysis of differences between atherosclerosis obliterans (AO) and diabetic macroangiopathy (DM) by examining relationships between variables and the probability of atherosclerosis occurrence in patients with one of these diseases

#### 3.4.1 Analysis of main effects

In order to assess the differences in qualitative and quantitative variables characterizing DM and AO, the odds of DM occurrence were analyzed in the group of patients with DM and AO. This approach allowed differentiation between the two conditions. The results of the analysis are presented in Table 2.

**TABLE 2:** Analysis of main effects.

Parameter	Studied category	Reference category	β_i_	β_i_ standard error	Wald statistic	p	Odds ratio (OR)	OR -95% CI	OR 95% CI
Sex	M	No	0.813	0.511	2,530	01,117	2,256	0.828	6,145
Polymorphism *PAR-1*: rs35900074 (506 I/D), rs32934 (1426 C/T), rs168753 (IVSn-14)	4	No	0.208	0.563	0.137	0.7116	1.232	0.408	3.715
Intercept		No	0.431	0.356	1.462	0.2266	1.538	0.765	3.093
*PAR-1:* rs35900074:I/D	8	No	−0.431	0.482	0.799	0.3713	0.650	0.253	1.671
Nicotine addiction	Yes	No	1.324	0.534	6.143	0.0132	3.759	1.319	10.714
vWF [%]	—	—	−0.023	0.009	6.927	0.0085	0.977	0.961	0.994
PDGF [pg/mL]	—	—	0.000	0.000	1.452	0.2283	1.000	1.000	1.000
TAT [µg/L]	—	—	−0.118	0.169	0.485	0.4861	0.889	0.638	1.239
IL-6 [pg/mL]	—	—	−0.159	0.161	0.983	0.3216	0.853	0.623	1.168
MCP-1 [pg/mL]	—	—	0.631	1.050	0.361	0.5477	1.880	0.240	14.710
sPECAM-1 [ng/mL]	—	—	−0.311	1.184	0.069	0.7929	0.733	0.072	7.464
TAFI [%]	—	—	−0.011	0.008	1.968	0.1606	0.990	0.975	1.004
PAF [µmol/min/mL]	—	—	0.446	0.377	1.401	0.2365	1.562	0.746	3.270
PLT [G/L]	—	—	0.004	0.004	1.508	0.2195	1.004	0.997	1.011
MPV [fl]	—	—	−0.366	0.211	2.999	0.0833	0.693	0.458	1.049
PT [%]	—	—	−0.040	0.030	1.810	0.1785	0.960	0.905	1.019
Fibrinogen [g/L]	—	—	1.324	1.234	1.151	0.2833	3.758	0.335	42.211
Total cholesterol [mg/dL]	—	—	−0.001	0.004	0.113	0.7362	0.999	0.990	1.007
HDL [mg/dL]	—	—	0.011	0.023	0.231	0.6310	1.011	0.966	1.058
LDL [mg/dL]	—	—	0.007	0.006	1.320	0.2506	1.007	0.995	1.018
Triglycerides [mg/dL]	—	—	−0.010	0.005	4.523	0.0334	0.990	0.980	0.999
hsCRP [mg/L]	—	—	0.004	0.062	0.005	0.9427	1.004	0.890	1.134
Glucose [mg/dL]	—	—	−0.251	0.168	2.222	0.1361	0.778	0.559	1.082
Urea acid [mg/dL]	—	—	0.213	0.288	0.548	0.4593	1.238	0.704	2.177
Total protein [g/dL]	—	—	0.213	0.288	0.548	0.4593	1.238	0.704	2.177
Platelet aggregation with ADP [%]	—	—	−0.005	0.016	0.108	0.7429	0.995	0.965	1.026
Platelet aggregation with collagen [%]	—	—	−0.007	0.026	0.064	0.8008	0.993	0.944	1.045
Platelet aggregation with TRAP [%]	—	—	−4.408	4.470	0.972	0.3241	0.012	0.000	77.756
Max aggregation time with TRAP [s]	—	—	−0.005	0.014	0.124	0.7249	0.995	0.968	1.023
Max aggregation time with ADP [s]	—	—	−0.017	0.016	1.223	0.2688	0.983	0.953	1.013
Max aggregation time with collagen[s]	—	—	0.001	0.008	0.011	0.9164	1.001	0.986	1.016
Microplatelets [%]	—	—	−0.040	0.021	3.821	0.0506	0.961	0.923	1.000
Activated microplatelets [%]	—	—	−0.052	0.020	6.663	0.0098	0.949	0.913	0.988
Aggregates of PLT [%]	—	—	−0.077	0.146	0.280	0.5964	0.925	0.695	1.233
Aggrgates of activated PLT [%]	—	—	−0.010	0.028	0.135	0.7129	0.990	0.936	1.046
PAR-1 [%]	—	—	−0.008	0.031	0.066	0.7972	0.992	0.933	1.055
PAR-1+TRAP [%]	—	—	−0.048	0.022	4.920	0.0265	0.953	0.913	0.994
Systolic blood pressure [mmHg]	—	—	−0.019	0.026	0.499	0.4798	0.981	0.932	1.034
Diastolic blood pressure [mmHg]	—	—	−0.005	0.037	0.019	0.8916	0.995	0.926	1.070
Age [years]	—	—	−0.127	0.044	8.406	0.0037	0.881	0.809	0.960
BMI [kg/m^2^]	—	—	−0.243	0.086	7.937	0.0048	0.784	0.663	0.929
ALAT [U/L]	—	—	0.011	0.017	0.458	0.4987	1.011	0.979	1.045
AspAT [U/L]	—	—	0.002	0.017	0.012	0.9135	1.002	0.970	1.035
GGTP [U/L]	—	—	0.002	0.006	0.135	0.7135	1.002	0.991	1.013
Urea [mg/dL]	—	—	−0.087	0.025	12.080	0.0005	0.917	0.873	0.963
Creatinine [mg/dL]	—	—	−0.632	1.563	0.164	0.6859	0.532	0.025	11.369

**FIGURE 7:**
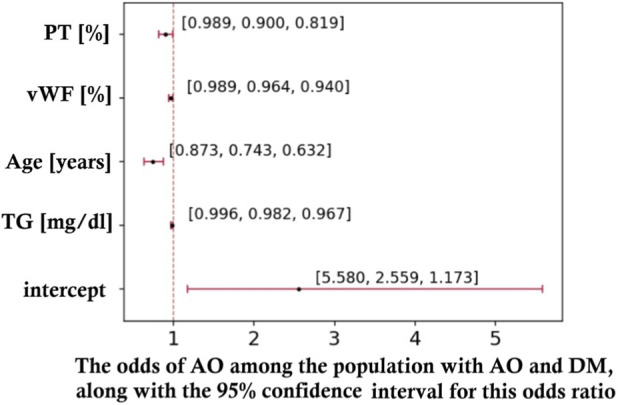
Multivariate impact of prothrombin time (PT, %), percentage of von Willebrand factor (vWF, %), age (years), and triglyceride concentration (TG, mg/dL) on the odds of developing atherosclerotic disease (AO) among the population of patients with either atherosclerotic disease or diabetic macroangiopathy (AO and DM).

The variables with a significant unidimensional impact on the differentiation between AO and DM were: smoking (p = 0.013), vWF concentration (p = 0.009), triglyceride levels (p = 0.033), relative number of activated microparticles (p = 0.010), PAR-1+TRAP concentration (p = 0.027), age (p = 0.004), BMI (p = 0.005), and urea concentration (p < 0.001). The odds ratio (OR) for the likelihood of AO *versus* DM (further referred to as “odds ratio”) was: 3.760 (smoking vs. non-smoking), 0.977 (for each 1 percentage point increase in vWF concentration), 0.990 (for each 1 mg/dL increase in triglyceride levels), 0.949 (for each 1 percentage point increase in the relative number of activated microparticles), 0.953 (for each 1 percentage point increase in PAR-1+TRAP concentration), 0.881 (for each 1-year increase in age), 0.784 (for each 1-unit increase in BMI), and 0.917 (for each 1 mg/dL increase in urea concentration). Subsequently, a multivariate model was developed using a stepwise method (Figure 3.12). The created multivariate model (iteratively, without interactions) utilized information about triglyceride levels (TG), patient age, von Willebrand factor (vWF) concentration, and prothrombin time (PT, %) to differentiate between AO and DM (Figure 7).

According to this model, among individuals with either of these diseases (AO or DM), the “baseline” odds of developing AO were 2.559 (reference patient: age 58 years, TG 126 mg/dL, vWF 154%, PT 104%). Each subsequent change by one unit of any of these variables resulted in a reduction in the odds by approximately: 1.83% (TG), 34.59% (age), 3.73% (vWF), and 11.11% (PT).

#### 3.4.2 Interaction analysis

**FIGURE 8:**
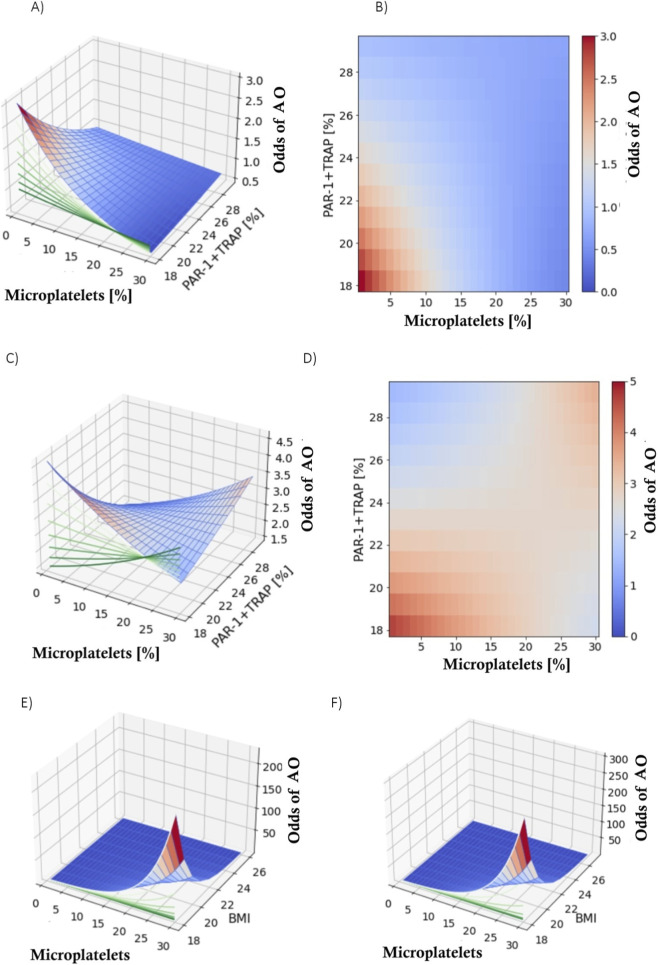
Multivariate analysis: **(A–D)** Number of microparticles with PAR-1+TRAP; **(E–F)** Number of microparticles with BMI (Figure 4.12E-F) and age with smoking.

To assess the existence of multifactorial differences between AO and DM, an interaction analysis was performed. In the first stage, interactions were selected that would significantly contribute information regarding the differentiation between the two conditions, compared to a naive model (not containing variables). The detailed results of this analysis are presented in Table 2. During the interaction analysis, the following second-order interactions were identified: the number of microparticles with PAR-1+TRAP (LR p = 0.006, in the factor model p = 0.020, Figures 8A–D), the number of microparticles with BMI (LR p = 0.012, in the factor model p = 0.029, Figures 8E–F), and age with smoking (LR p = 0.031, in the factor model p = 0.035).

In a patient with a relative platelet microparticle count of 10.90%, an increase in PAR-1+TRAP1 by 1 percentage point caused a 5.93% decrease in the likelihood of developing AO. Although the increase in platelet microparticles (by 1 percentage point) did not significantly change this likelihood on its own (p = 0.103), it modulated the change in likelihood caused by the increase in PAR-1+TRAP1 by 1.005 times (Figures 3.13A–D; Table 2). In a patient with a platelet microparticle percentage of 10.90%, an increase in BMI by 1 unit led to a 24.84% decrease in the likelihood of developing AO. Although the increase in platelet microparticle percentage (by 1 percentage point) did not significantly change this likelihood on its own (p = 0.162), it modulated the change in likelihood caused by the increase in BMI by 1.022 times (Figures 3.13E–F; Table 2). In a 58-year-old patient, smoking was associated with a 3.352-fold increased likelihood of developing AO. Among non-smokers, with each year of age, this likelihood decreased by 22.10%. The increase in one parameter modulated the impact of the other on this likelihood by 1.22 times (or 22%).

## 4 Discussion

Atherosclerosis obliterans (also referred to as lower limb atherosclerosis) is a form of peripheral artery disease (PAD). Symptoms such as rest pain, non-healing ulcers, and gangrene caused by chronic ischemia (Fontaine stage III–IV or corresponding Rutherford categories) are classified as chronic limb-threatening ischemia (CLTI), also known as critical limb ischemia (CLI) (Ye et al., n.d.). This condition severely impacts quality of life and functional capacity, often resulting in death (Iso et al., 2010). Due to the extent of disease, anatomical lesion location, or significant comorbidities, revascularization is not an option for up to 40% of patients. In such cases, amputation remains the only therapeutic solution, though it is associated with poor outcomes: perioperative mortality ranges from 5% to 20%, a second amputation is needed in 30% of cases, and only 25%–50% of patients regain full mobility (Iso et al., 2010). DM is one of the most significant comorbidities that exacerbates and accelerates atherosclerosis. Chronic inflammation driven by hyperglycemia, the accumulation of advanced glycation end products (AGEs) in vessel walls, and elevated oxidative stress initiates atherogenic changes throughout the arterial system. The diffuse nature of diabetic macroangiopathy is due to the vast surface area of the peripheral vascular bed, contributing to the progression of additional complications such as coronary artery disease, cerebrovascular conditions, neurological disorders, and CLTI. In diabetic patients, PAD risk increases with age, diabetes duration, and peripheral neuropathy, stemming from vascular inflammation, blood element dysfunction, and hemostatic abnormalities (Thiruvoipati et al., 2015; Polpili and AuthorAnonymous, 2019). The progression of both AO and DM is often severe, leading to similar clinical outcomes (e.g., limb ischemia, coronary artery disease, stroke), yet their etiologies and pathophysiological mechanisms differ. Therefore, there is a critical need to identify early, condition-specific biomarkers. Understanding these mechanisms not only improves the diagnosis of atherosclerosis but may also support the discovery of new therapeutic targets and help delay the onset of clinical symptoms. However, there is still a lack of molecular tools capable of pinpointing key drivers of progression in specific stages of DM or AO. It is well established that numerous biochemical factors contribute to atherosclerosis, including inflammatory mediators, modified LDL, blood elements, oxidative stress products, and glycation products. The complexity of the condition stems from the large number of molecules and pathways involved. Classifying atherosclerosis into subtypes like AO and DM helps organize this heterogeneity based on etiology.

Current research focuses on identifying strong associations between anthropometric, laboratory, and imaging parameters and the development of AO and DM, while also exploring links between these conditions and novel biomarkers involved in critical stages of atherogenic progression. These include platelet activation, inflammation onset and progression, and co-existing disorders of hemostasis, coagulation, and fibrinolysis. Evaluating the role of specific metabolites—their levels and activity—offers valuable insights into the pathophysiological mechanisms driving various forms of atherosclerosis.

PLT play a crucial role in the mechanisms of atherosclerosis progression–although it is known that both their activation (e.g., by thrombin) and aggregation exhibit atherogenic effects, the significance of these processes in atherosclerosis of different etiologies, such as DM or AO, is still not fully understood. To date, it has been shown that the initiation and development of atherosclerosis through the activation of factors inducing PLT adhesion to the endothelium involves the interaction of PARs (protease-activated receptors) with thrombin–the expression of PAR-1 increases as atherosclerosis progresses (Ossovskaya and Bunnett, 2004), which is associated with enhanced thrombin secretion. These receptors are considered key therapeutic and diagnostic targets that could slow down atherogenic processes, due to their molecular properties. PARs are transmembrane receptors coupled with G proteins, uniquely activated by proteolytic cleavage of their extracellular portion.

This cleavage “unmasks” a new N-terminus, which acts as a “tethered” ligand, binding to the second extracellular domain of the protein and causing various cellular responses. Physiologically, PAR-1 is expressed in various tissues, including on the surface of endothelial cells, neurons, fibroblasts, and epithelial cells. However, PAR-1 overexpression has been observed in some metabolic disorders, such as atherosclerosis, and in various human cancers, e.g., on the surface of breast, colon, prostate, esophageal, ovarian cancer cells, as well as in melanoma and aggressive leukemias (including in crisis phases), where it is a negative prognostic factor (Hagag et al., 2014). PAR-1 also plays a role in several pro-cancer reactions–for example, it is associated with primary growth, aggressive invasiveness, metastasis, and angiogenesis (Hagag et al., 2014). Furthermore, it is known that PAR-1 is a receptor present on endothelial cells, smooth muscle cells, and on the surface of morphotic elements, such as leukocytes (LEU) and PLT. Activated platelets induce an increased synthesis of chemokines, which is closely related to the increased expression of adhesion molecules, primarily E-selectin, VCAM-1, and ICAM-1 (Jakobsche-Policht et al., 2014). PAR-1 contributes to vascular disorders by mediating the cellular response triggered by thrombin and related proteases. An agonist of the PAR-1 receptor is also TRAP (thrombin receptor-activating peptide) – a substitute for thrombin. Activation of PAR-1 by thrombin or TRAP leads to activation of GPIIb/IIIa receptors, enabling platelets to bind to von Willebrand factor, which is essential for platelet adhesion to collagen. However, it is still not fully understood in which subtype of atherosclerosis platelet activation through the interaction of PAR-1 with thrombin plays a key role.

The conducted studies showed that the level of PAR-1 expression on PLT that were not exposed to TRAP was comparable in the plasma of patients with AO, DM and the control group. However, the application of TRAP resulted in a significant increase in PAR-1 levels on platelets in patients with DM, not only compared to platelets from the control group but also compared to patients with AO. These results suggest that the interaction between platelet PAR-1 and thrombin may play a crucial role in the initiation and development of atherosclerosis, primarily in the context of DM. However, this observation should be confirmed through studies involving a significantly larger number of platelet-rich plasma samples from patients with DM and AO. Furthermore, the statistical analysis, considering gender, revealed significantly higher PAR-1 expression on platelets in men with AO compared to women with AO and significantly lower expression compared to men with DM. This suggests that platelets in men with AO are significantly more predisposed to activation involving thrombin than platelets in women with AO, though this activation is still significantly lower than in men with DM. Given that the interaction of platelet PAR-1 with thrombin induces platelet shape changes and initiates the release of Ca^2+^ and other compounds from cytoplasmic granules, thus increasing platelet aggregation properties, many studies suggest that this initial stage of the cascade leading to platelet aggregation is crucial (Jakobsche-Policht et al., 2014), and PAR-1 serves as both a diagnostic and therapeutic target.

Currently, several molecular tools have already been introduced that inhibit or even prevent the interaction between PAR-1 and thrombin (Liu et al., 2017). For example, there are antibodies that interfere with proteolysis, thereby preventing receptor activation (Hung et al., 1992; Cook et al., 1995). The epitopes for these antibodies are proteins encoded by the exodomain sequences of PAR-1, such as the sequence encoding the thrombin proteolysis site and the sequence encoding the hirudin-like fragment (Hung et al., 1992). It is also possible to orthosterically disrupt the interaction between the “bound” ligand (the new N-terminal formed as a result of thrombin action) and the ligand-binding site through the use of peptide mimetics, such as vorapaxar (SCH-530348), a PAR-1 antagonist. In preclinical studies, vorapaxar demonstrated antiplatelet activity, inhibiting *in vitro* aggregation of human platelets induced by 10 nM thrombin or 15 µM high-affinity thrombin receptor-activating peptide (haTRAP) (Chackalamannil et al., 2008). Currently, vorapaxar has been introduced as the first drug in the class of PAR-1 antagonists, which can be used by patients with a positive history of myocardial infarction or peripheral artery disease (PAD), even in combination with aspirin and/or clopidogrel, to reduce cardiovascular risk in these patients (French et al., 2015). Due to the fact that vorapaxar is contraindicated in patients with a history of stroke, transient ischemic attack, intracranial hemorrhage, and in patients weighing less than 60 kg (due to the increased risk of brain bleeds, which potentially outweigh the therapeutic benefit), further research is needed to explore new PAR-1 antagonists (Frampton, 2015). Blocking the effects of the interaction between thrombin and PAR-1 can also occur at the stage of inhibiting the recruitment of G proteins to the third intracellular loop (ICL3) of PAR-1, for example, through pepducins (Covic et al., 2002a; 2002b). These structures, penetrating palmitoylated peptides, can be designed to fit sequences such as PAR-1 (pepducin P1pal-12, P1pal-7) or PAR-4 (pepducin P4pal-10). Based on *in vitro* studies, it has been found that pepducins can be incorporated into the cell membrane, creating an area corresponding to the interaction between the G protein and GPCR, thus disrupting cellular signaling. For example, P1pal-12 removes free G protein from the cytosol, which interferes with the recruitment of G proteins to PAR-1 (Covic et al., 2002b). Currently, P1pal-7 has already been introduced as a drug (PZ128) for anticoagulation in the treatment of non-acute conditions within coronary percutaneous interventions (TRIP-PCI) (Rana et al., 2018).

It has also been observed that compounds such as parmodulins, which are targeted at the cytoplasmic surface of PAR-1 (without modifying the endogenous ligand-binding site on the extracellular side), selectively block the intracellular calcium flow mediated by the PAR-1–Gq axis. This action inhibits platelet aggregation but does not affect platelet shape changes–the activity of RhoA through the PAR-1–G13 axis, which determines platelet shape changes, remains unchanged. It is also known that activation of PAR-1 located on the surface of endothelial cells and smooth muscle cells contributes to vascular constriction and narrowing, as well as leads to the release of von Willebrand factor (vWF), endothelin, nitric oxide (NO), and prostacyclin (PGI2), which may be associated with an increase in cyclic adenosine monophosphate (cAMP) levels (Jennings, 2009). So far, it has been demonstrated that, apart from inhibiting platelet activity in thromboembolic disease models both *in vitro* and *in vivo*, PAR-1 antagonists serve as probes for studying PAR-1 activation mechanisms involving the “bound” ligand (Hamilton and Trejo, 2017). Additionally, in the endothelium, parmodulins inhibit the pro-inflammatory effect mediated by PAR-1 without affecting the protective APC pathway (Aisiku et al., 2015). Confirmation of the significant role of platelet activation, particularly in DM, through studies on a larger group of patients than in the presented study, may lead to the introduction of existing drugs that inhibit the PAR-1/thrombin interaction into pharmacotherapy at the early stages of DM.

Most of the genes encoding receptors present on PLT have already been sequenced, allowing for the identification of polymorphisms occurring in both coding and regulatory regions—areas of the gene where mutations most commonly arise. Ongoing research conducted by various teams continues to expand our understanding of the consequences of these polymorphisms for platelet function and their role in predisposing individuals to excessive bleeding or thrombosis. Typically, high receptor density is associated with an increased risk of thrombosis, whereas low receptor density is linked to excessive bleeding (Dupont et al., 2003a). For example, differences in the density of the α2β1 integrin on PLT surfaces in healthy individuals have been shown to correlate with the degree of platelet adhesion to collagen. Variations in receptor density may not only result from an ongoing pathological process but can also stem from mutations in the genes encoding these receptors. It has been observed that the expression levels of PAR-1 on PLT show interindividual variability in receptor numbers while maintaining stable receptor density over time, indicating the presence of genetic mutations in the PAR-1 gene (Cadroy et al., 2001). Currently, three known polymorphisms exist in the gene encoding PAR-1. Two of these are located in the 5′regulatory region: a C-to-T transition at 1,426 base pairs (bp) upstream of the transcription start site (1426 C/T) and a 13-bp insertion (I) of the preceding sequence 5′-CGGCCGCGGGAAG-3′ (506 I/D). The third polymorphism is an A-to-T transversion located in an intervening sequence (IVS), 14 nucleotides upstream of the exon 2 start site (IVSn 14 A/T). The frequency of mutations in a given segment of the PAR-1 gene varies across different disease conditions. In healthy individuals, the estimated allele frequencies for IVS-14T, 1426T, and −506I are 0.185, 0.041, and 0.256, respectively (Arnaud et al., 2000). A similar frequency for the −506I/D polymorphism in healthy individuals was reported by another research team as 0.27 (Afife Karabiyik, 2011). In the present study, the observed allele frequencies for IVSn-14 T, 1426 T, and 506 I were 0.194, 0.028, and 0.27, respectively. The frequency of the T allele in the control group was lower at 0.028, which may have resulted from the relatively small sample size. The 13-bp insertion polymorphism, designated as −506 I/D (F2R, rs35900074), is not associated with the platelet PAR-1 phenotype (Dupont et al., 2003b). In this study, three variants of the −506 I/D polymorphism in the PAR-1 gene were identified: the I/I homozygous genotype, the I/D heterozygous genotype, and the D/D homozygous genotype. The highest percentage of the −506 I/D heterozygous variant was observed in the AO group. The 506 I/I variant was rare, whereas the −506 D/D homozygous genotype was predominant across all groups, including DM, AO, and the control group. These findings suggest that the presence of the heterozygous I/D variant at position −506 I/D in the PAR-1 gene may be associated with a reduced susceptibility to platelet PAR-1 activation by thrombin. Consequently, this mutation–along with the chronic exposure of platelets to hyperglycemia in DM–may contribute to a slower progression of atherosclerotic processes in AO compared to DM. These findings were further confirmed in another study presented in this work, which aimed to determine the level of PAR-1 expression activated by TRAP in relation to different genotypes (DD, I/D, and II). It was observed that the expression level of PAR-1 in DM patients was significantly higher in individuals with the DD genotype, particularly among men. This suggests that the −506 DD polymorphism has a significant impact on increasing PAR-1 gene expression in patients with both DM and AO. This observation aligns with conclusions drawn from another study, which demonstrated that the I allele in I/I homozygotes is less common in men with thromboembolic disease than in the male control group. Additionally, a reduction in prothrombin fragment F1+2 levels was found in homozygous carriers of the −506 I allele. These data suggest a potential protective effect of the −506 I insertion polymorphism in men affected by venous thromboembolism (Arnaud et al., 2000). Moreover, it has been shown that the D allele is associated with better survival prognosis and a lower risk of disease recurrence in female breast cancer patients (Eroǧ;lu et al., 2012). Additionally, the DD genotype is significantly more prevalent in healthy individuals than in the patient group, which is consistent with the findings of this study–where a significantly higher frequency of the D allele was observed in the control group compared to the AO or DM patient groups (Afife Karabiyik, 2011). The −1426 C/T polymorphism (F2R, rs32934), similar to the −506 I/D polymorphism, is not associated with the platelet PAR-1 phenotype. Additionally, studies conducted by various research teams indicate that the occurrence of the T allele in place of the C allele is very rare (0.041). In the present study, a significant number of samples exhibited only the C/C homozygous genotype, while the −1426 C/T polymorphism was observed only once. This finding aligns with observations from other research teams, suggesting that in a small study group, individuals with the CT or TT genotype may not be present at all. The IVS-14 A/T single nucleotide polymorphism (SNP) in the PAR-1 gene may influence receptor density on PLT and their function in healthy individuals (Dupont et al., 2003b). Moreover, an association has been demonstrated between the presence of the T and A genotypes and the progression of liver fibrosis in chronic hepatitis C (Martinelli et al., 2008). A trend toward a higher rate of fibrosis progression was observed in patients with the TT genotype (P = 0.06), and the AA genotype was associated with slow fibrosis progression (P = 0.03) in the European population. Additionally, in men, the rate of fibrosis progression was significantly higher in individuals with the TT genotype with heterozygotes (P = 0.003) and AA (P = 0.007). A significant association between the TT genotype and rapid fibrosis progression was identified (P = 0.04). Importantly, this finding was confirmed in an independent Brazilian cohort, where the rate of liver fibrosis progression was also higher in individuals with the TT genotyp (P = 0.03). Moreover, no differences were observed between the IVS-14 A/T and −506 I/D genotypes and the progression of liver fibrosis. The T allele (with an allele frequency of 0.14) is associated with lower PAR-1 expression on PLT surfaces and a reduced PLT response to activation by the SFLLRN peptide (an agonist of the PAR-1 amino terminus) compared to the A allele (Dupont et al., 2003b). Furthermore, differences in PLT responses to SFLLRN in healthy individuals correlate with variations in PAR-1 density on the PLT surface, and this variability is at least partially inherited through certain PAR-1 polymorphisms (Dupont et al., 2003a). It has also been demonstrated that the number of PAR-1 receptors on PLT is linked to intronic variability in the IVSn-14 A/T sequence, confirming its role in regulating PLT responses to SFLLRN in terms of aggregation and P-selectin expression. This intronic variability may, therefore, have clinical significance. Animal studies using PAR-1 antagonists suggest that reduced PAR-1 availability may increase the tendency to bleed. Consequently, inherited differences in receptor density could be clinically relevant in individuals already at an increased risk of bleeding, such as those with mild hemophilia or von Willebrand disease, as well as patients undergoing surgical procedures. The multivariate Cox regression analysis revealed that carrying the T allele in the PAR-1 gene is an independent predictive factor for composite ischemic events, even after accounting for other risk factors (Zhang et al., 2016). The risk of ischemic events is lower in T allele carriers. However, studies conducted on plasma samples from T allele carriers (allele frequency of 14.8%) to assess the potential reduction in thrombotic event risk and increased bleeding risk after PCI (for unstable or stable coronary artery disease) did not show a significant association between genotype differences and cardiovascular events (33.7%, 28.8%, and 31.6% for A/A, A/T, and T/T genotypes, respectively; p = 0.50) or bleeding (15.7%, 14.7%, and 18.8% for A/A, A/T, and T/T genotypes, respectively; p = 0.90). In the Cox regression model adjusted for age, race, sex, BMI, and smoking status, T allele carriage was not significantly associated with cardiovascular events (HR 1.19, 95% CI 0.89–1.59, p = 0.23) or bleeding (HR 0.73, 95% CI 0.37–1.4, p = 0.34) (Friedman et al., 2016). However, in STEMI patients undergoing PCI, the presence of at least 1 T allele was associated with a significantly lower risk of ischemic syndrome compared to homozygous A allele carrier (Smith et al., 2005). Additionally, the IVSn 14 A/T intronic polymorphism may influence mRNA processing speed or enhance transcription efficiency and the amount of synthesized protein (Dupont et al., 2003b). It has been shown that the presence of the T allele is associated not only with lower PAR-1 expression on the surface of PLT but also with reduced platelet aggregation, decreased cellular response to the PAR-1 activating peptide, and lower procoagulant activity. The potential link between the IVS-14 A/T polymorphism and the platelet PAR-1 phenotype may be due to its effect on intron splicing. The sequence variation occurs in an intervening sequence (IVS), 14 nucleotides upstream of exon 2’s start site, which may influence splice site recognition. However, no sequence differences were detected in the exon 2 junction region on platelet cDNA among individuals homozygous for the A allele, heterozygous, or homozygous for the T allele (Dupont et al., 2003b). PAR-1 expression may also be regulated at the transcriptional level. However, quantitative gene expression analysis at the mRNA level did not show any correlation with the number of PAR-1 receptors or the IVS-14 A/T intronic polymorphism (Dupont et al., 2003b). It has been observed that homozygous carriers of the A allele exhibit increased PLT aggregation and a heightened procoagulant response. However, clopidogrel effectively inhibits this response regardless of the PAR-1 genotype. Notably, individuals with the A allele more frequently exhibit persistent high PLT reactivity despite clopidogrel treatment, which increases the risk of thromboembolic events and necessitates additional antiplatelet therapy. In the present study, similar trends were observed regarding the distribution of homozygous A/A and heterozygous A/T individuals, aligning with previous research findings. The percentage of A/A homozygotes was the highest in all studied groups, while A/T heterozygotes appeared less frequently and at a comparable rate across all groups. Meanwhile, the T/T homozygote was very rare. Additionally, the A/A homozygote in the IVS-14 polymorphism of the PAR-1 gene was significantly more prevalent in patients with DM and AO compared to the control group. This finding suggests a stronger predisposition to PLT activation and aggregation in DM and AO patients compared to healthy individuals.

Moreover, associations between the A/T genotypes of the PAR-1 gene and various diseases have been reported. For example, in chronic obstructive pulmonary disease (COPD), the A allele (A/A) has been linked to a higher risk of developing the disease. Polymorphism analyses were conducted in a group of 270 COPD patients and 270 control subjects using polymerase chain reaction–restriction fragment length polymorphism (PCR-RFLP) analysis. The frequency of the AA genotype in the PAR1 IVS-14 A/T polymorphism (rs168753) was significantly higher than in the control group (Yun and Sang, 2015). Furthermore, given that carriers of the T allele in IVS-14 exhibit reduced platelet reactivity and a lower risk of thrombotic events, it was hypothesized that they might have a tendency to bleed, for example, following percutaneous coronary intervention (PCI). However, studies conducted by Friedman et al. showed no such association, indicating that this hypothesis is incorrect. Another research group reported a correlation between the presence of A/T heterozygotes and recurrent pregnancy loss (Grisaru-Granovsky et al., 2015). These isolated findings currently serve only as preliminary indications for further research on the impact of PAR-1 gene polymorphisms on the molecular mechanisms involving its encoded receptor. Nevertheless, they are not insignificant, as they provide a direction for ongoing studies.

In the present study, PAR-1 mRNA levels did not correlate with receptor abundance. The level of PAR-1 mRNA represents only one of the factors regulating protein expression. The number of receptors on the cell surface also depends on post-translational modifications, protein stability, and internalization mechanisms. Even with high mRNA expression, translation can be limited by microRNAs, translation-inhibiting proteins, or restricted ribosome availability. Moreover, PAR-1 is rapidly internalized and degraded upon activation, and polymorphisms can affect protein stability or trafficking independently of transcript levels. Environmental factors, such as oxidative stress, cytokines, or growth factors, can further modulate the number of surface receptors. Consequently, the lack of correlation between mRNA levels and receptor numbers is not surprising.

The activation of PLT initiated by the interaction of PAR-1 with factor Xa or thrombin is a crucial process determining the development of atherosclerosis. It influences both the formation and progression of atherosclerotic plaques, as well as the initiation and amplification of the associated inflammatory response (both local and systemic) (Borensztajn et al., 2009). It has been demonstrated that thrombin, through the proteolytic activation of PAR-1, PAR-3, and PAR-4 receptors, precisely regulates both physiological and pathological vascular states (Hirano, 2007). The activation of the PAR-2 receptor initiates pro-atherogenic processes such as oxidative stress induction, monocyte and fibroblast chemotaxis, inflammation, migration and proliferation of vascular smooth muscle cells (VSMCs), angiogenesis, and apoptosis (Monroe and Key, 2007). As a result of the pathological mechanisms triggered by thrombin’s interaction with PAR receptors, the synthesis of inflammatory cytokines such as IL-6 and IL-8, and type 2 chemokines like CCL-2, as well as the expression of E-selectin, ICAM-1, VCAM-1, TF, and the proliferation of VSMCs with the release of growth factors, is initiated (Borensztajn et al., 2009). Many coagulation system proteins contribute to endothelial barrier damage, oxidative stress development, leukocyte recruitment, inflammation, migration and proliferation of VSMCs, immune responses, apoptosis of PLT and other cells, and the acceleration of angiogenesis-related processes (Borensztajn et al., 2009; Borissoff et al., 2011). Increased thrombin production in the early stages of atherosclerosis also stimulates PLT and the endothelium to upregulate PAR-1 expression. Activated PLT then interact with the endothelium *via* the interaction of platelet P-selectin with a glycoprotein ligand present on endothelial cells (EC), known as P-Selectin Glycoprotein Ligand-1 (PSGL-1). This interaction is further stabilized by β3 glycoprotein. The activation of PLT is influenced by coagulation-related factors such as the TAT, (TAFI, vWF. TAT is considered a sensitive marker of coagulation activation (so-called “prothrombotic readiness”) – its concentration measurement is assumed to reflect thrombin expression (Song et al., 2023). TAFI, on the other hand, weakens fibrinolysis mechanisms and promotes thromboembolic complications. A significant increase in serum TAT levels has been observed in patients with chronic peripheral arterial insufficiency due to atherosclerosis (Gosk-Bierska et al., 2016). Similarly, in the present study, significantly higher levels of TAT and TAFI were found in patients with both DM and AO compared to the group of healthy volunteers, indicating a higher “prothrombotic readiness” in these patients. Gender analysis revealed that in the group of men with AO, TAT levels were significantly higher than in healthy men, while in the group of men with DM, both TAT and TAFI levels were significantly higher than in healthy volunteers. Additionally, the study showed that in both types of atherosclerosis: DM and AO, vWF levels were significantly higher than in the control group. Interestingly, only the difference in vWF levels was significantly lower in AO patients compared to DM patients. This observation suggests that vWF may be an important factor distinguishing obstructive atherosclerosis from DM. The significantly lower release of vWF (in the form of granules) from PLT in AO compared to DM, as demonstrated in this study, may indicate reduced PLT adhesion to the endothelium, which consequently leads to inflammation attenuation by decreasing the number of factor VIII molecules—a phase-acute protein transported by vWF. The results presented in this study align with findings from other research groups, which indicate significantly higher vWF concentrations in diabetic patients at risk of cardiovascular diseases compared to healthy, physically active individuals (Fiodorenko-Dumas et al., n.d.), as well as in individuals with atherosclerosis compared to healthy individuals (Gosk-Bierska rt al., 2002).

Moreover, PAR-1-dependent platelet activation may be modulated by the localization of these receptors within lipid rafts, which can influence signal transduction efficiency and the severity of vascular pathologies. PAR receptors, as members of the GPCR family, are preferentially localized within membrane microdomains enriched in cholesterol and sphingolipids, which serve as signaling platforms (Riitano et al., 2022). These microdomains facilitate receptor and signaling protein clustering, and the localization of PAR-1 and PAR-2 within lipid rafts enhances their interactions with kinases, adaptor proteins, and other membrane receptors, thereby increasing downstream signaling efficiency. Within these domains, receptors can form clusters that enhance their activation probability, cellular response initiation, and sensitivity to ligands such as thrombin in the case of PAR-1. Emerging evidence indicates that protease-activated receptors, including PAR-2, are functionally organized within lipid rafts alongside other membrane proteins, promoting prothrombotic signaling. Notably, PAR-2 forms a complex with the co-receptor LRP6 and β2-GPI in endothelial cell lipid rafts upon stimulation with anti-β2-GPI antibodies (Riitano et al., 2022). Co-fractionation in Triton X-100-resistant membranes and immunoprecipitation confirmed the physical association of these proteins. Complex activation induced phosphorylation of LRP6 and β-catenin, whereas raft disruption by methyl-β-cyclodextrin significantly attenuated signaling, highlighting the functional dependence on microdomain integrity. Similarly, Nag et al. (2017) demonstrated that PAR-2 stimulation with the specific agonist SLIGKV promotes physical association with LRP6, phosphorylation of LRP6, Axin recruitment, and β-catenin stabilization, resulting in Wnt/β-catenin pathway activation, a process critically dependent on receptor organization within lipid rafts. The importance of raft integrity for PAR-2–LRP6 signaling and associated prothrombotic outcomes has been further emphasized in recent reviews (Capozzi et al., 2023), where raft disruption reduced tissue factor expression. Computational modeling also indicates that the receptor-to-raft ratio dictates LRP6 phosphorylation efficiency and downstream Wnt/β-catenin signaling (Haack et al., 2021). Additional evidence confirms that LRP6-dependent signaling is generally raft-dependent, as microdomain disruption impairs β-catenin phosphorylation induced by tPA (Riitano et al., 2020), and LRP6 internalization following oxidized phospholipid binding requires caveolin, consistent with its raft localization (Wang et al., 2018). In platelets, which are particularly enriched in lipid rafts and release numerous microparticles, PAR localization within these microdomains may regulate both platelet activation and the secretion of prothrombotic and proinflammatory mediators, central components of thrombo-inflammatory processes. Given the parallels with PAR-2, it is plausible that PAR-1-mediated platelet activation similarly depends on raft localization, potentially determining thrombin-induced signaling efficiency and influencing the progression of vascular diseases such as atherosclerosis and diabetes. Collectively, these data suggest that both the distribution and activation of PAR-1 are likely regulated by lipid raft localization, and that microplatelets or microparticles derived from these regions may modulate thrombo-inflammatory responses in atherothrombosis and diabetes. Investigation of this phenomenon could provide mechanistic insights into receptor accessibility, clustering, and signaling in vascular pathophysiology.

The interaction of PAR-1 with thrombin also induces the expression of MCP-1 in EC and VSMCs (Libby and Theroux, 2005). MCP-1 is a chemokine that, by interacting with its receptor CCR2 (Chemokine Receptor Type 2), stimulates monocytes to transmigrate through EC junctions and the basement membrane, exacerbating endothelial dysfunction (Libby and Theroux, 2005). In the present study, a significant increase in MCP-1 concentration was observed in the group of patients with DM and AO compared to the control group. Moreover, gender-based analysis revealed that both women and men with AO or DM exhibited significantly higher MCP-1 levels than the control group. The elevated MCP-1 levels found in this study are consistent with results published by other research groups, which indicate an intensification of inflammatory processes involving MCP-1 in patients with atherosclerosis compared to individuals without AO or DM (Li et al., 2020; Desaki et al., 2023). Activated monocytes and macrophages can subsequently synthesize, release, and expose tissue factor (TF), phospholipids (PL), PAF, other proinflammatory cytokines (IL-1, IL-8, TNF), leukotrienes, thromboxanes, and toxic oxygen metabolites (Shibata et al., 2008). Under physiological conditions, the endothelial lining does not express TF; however, its expression increases in adjacent layers under pathological conditions. In atherosclerotic plaques, TF is present in macrophages, VSMCs, and the remnants of foam cells within the necrotic core of the plaque (Borissoff et al., 2010).

Extracellular vesicles (EVs) are small vesicles released by cells through processes such as exocytosis, plasma membrane budding, or apoptosis. Depending on their biogenesis, they are classified as exosomes (50–150 nm), microparticles (100–1,000 nm), or apoptotic bodies (Boilard et al., 2015). Microparticles are secreted by platelets, leukocytes, erythrocytes, and endothelial cells. They contain membrane proteins, enzymes, lipids, and genetic material, participating in the regulation of inflammation, neovascularization, coagulation, and fibrinolysis. Their cellular origin can be identified based on surface markers, e.g., CD42a for platelets, CD45 for leukocytes, or CD144 for endothelial cells (Barteneva et al., 2013). Platelet-derived microparticles (PMPs) are formed upon platelet activation or apoptosis and act as key procoagulant and proinflammatory mediators. Their release is triggered by stimuli such as collagen, thrombin, ADP, PAF, oxidative stress, or shear forces. This process involves intracellular Ca^2+^ elevation, calpain activation, and externalization of phosphatidylserine to the outer membrane leaflet (Varon and Shai, 2015; Konkolewska et al., 2014). PMPs contain platelet membrane proteins (CD41, CD42b, CD62P), coagulation factors (Va, VIII), chemokines, cytokines, microRNAs, and phosphatidylserine, enabling the assembly of coagulation complexes. They interact with leukocytes and endothelial cells, modulating proinflammatory, procoagulant responses, angiogenesis, and tumor progression (Boilard et al., 2015; Kassassir et al., 2023). PMPs exhibit strong procoagulant activity, surpassing that of intact platelets, through phosphatidylserine exposure and coagulation factor transfer. They can also support immune responses and exert anticoagulant functions, e.g., *via* protein C activation. Their levels are elevated in pathological conditions such as stroke, myocardial infarction, disseminated intravascular coagulation (DIC), systemic lupus erythematosus, and antiphospholipid syndrome, making them potential biomarkers for inflammatory, cardiovascular, and neoplastic diseases (Rother et al., 2022; Varon and Shai, 2015; Jakobsche-Policht et al., 2025). In summary, microparticles, including PMPs, are critical regulators of coagulation, inflammation, angiogenesis, and tumor progression, representing valuable diagnostic and therapeutic targets in translational medicine. The analysis of interactions occurring in the studied group of patients (AO and DM) revealed that individuals with a low percentage of microparticles and simultaneously low activation of PAR-1 (present on platelets, including microparticles) *via* TRAP have a significantly higher chance of AO. This means that platelet activation (with a high concentration of microparticles) by thrombin is more frequently observed in patients with DM. The same analysis (Figures 8C and D), which inferred conclusions from the population from which the studied group of patients originated, showed that it is not the number of microparticles themselves, but rather their relationship with PAR-1 activation by TRAP that influences the chance of AO. In individuals with intermediate PAR-TRAP values (20%–26%), even with a high percentage of microparticles, an increased (about 2-fold) chance of AO is observed. The lowest chance of AO is observed for PAR-1 + TRAP >26% and microparticle percentage <15%.

## 5 Summary

Significantly lower values of BMI, glucose concentration, and urea concentration, as well as significantly lower values of the claudication distance coefficient, were observed in the group of patients with occlusive atherosclerosis compared to the group of patients with diabetic macroangiopathy. PLT from patients with diabetic macroangiopathy showed a higher predisposition for the initiation and development of atherosclerotic disorders accompanied by PAR-1 activation than platelets from patients with occlusive atherosclerosis. It was shown that mutations in the PAR-1 gene (−506 D/D; IVS-14 A/A), which favor platelet activation and aggregation, were significantly more frequent in the diabetic macroangiopathy group. In contrast, in the occlusive atherosclerosis group, despite the frequent presence of the homozygous IVS-14 A/A, PAR-1 expression was lower, possibly due to the more frequent presence of the heterozygous −506 I/D. This indicates the protective properties of the I allele. Microplatelets activation using TRAP proved to be a useful method in differentiating patients with occlusive atherosclerosis from those with diabetic macroangiopathy. However, this observation should be confirmed by conducting studies on a significantly larger group of patients. Significantly lower levels of vWF were found in the group of patients with occlusive atherosclerosis compared to the group of patients with diabetic macroangiopathy. Consequently, this contributes to a slower progression of atherosclerotic and inflammatory processes–lower levels of vWF reduce both platelet adhesion to the endothelium and the number of factor VIII molecules (an acute-phase protein) carried by vWF. Furthermore, interaction analysis showed that although the percentage of microplatelets did not affect the likelihood of AO (among patients with AO or DM), at high values of the microparticle percentage (above 23%), the intensity of PAR-1 activation by TRAP (a thrombin substitute) became a factor increasing the likelihood of AO.

## 6 Limitations and further plans

The conducted studies indicate that the presence of at least one I allele at position D in the 506 region of the PAR-1 gene may be a significant prognostic factor for the severity of atherosclerosis. We acknowledge the limitations of this publication, such as the small study groups. However, the research was of a preliminary nature. It was crucial to conduct the broadest possible spectrum of various laboratory studies using different techniques, including flow cytometry, molecular studies, immunoenzymatic tests, and classical laboratory and clinical methods commonly used in hospital practice. This approach enabled the search for potential markers of atherosclerosis severity. The proposed original method for detecting activated microparticles also proved valuable, suggesting that microparticle count itself may serve as a potential prognostic indicator of atherosclerosis.

In the next stage of the study, we plan to examine a significantly larger population, including healthy individuals, those with varying degrees of obstructive atherosclerosis, and individuals with different stages of diabetic macroangiopathy. Biological material will be collected from these individuals at multiple follow-ups over a 2-year interval. In addition to determining PAR-1 polymorphisms, levels of activated microparticles, and hemostatic markers through laboratory and imaging diagnostics, the collected biological material will also undergo proteomic and lipidomic analyses. This will allow for detailed multidimensional analyses and, ultimately, the development of prognostic algorithms. However, given the rapidly increasing number of patients with type 2 diabetes complicated by macroangiopathy, we believe that our findings could provide a valuable research direction for other scientific teams.

## Data Availability

The original contributions presented in the study are publicly available. This data can be found here: 10.60956/66mf-mf09.
