# Supplementary material for: The predictive role of protease-activated receptor (PAR-1) polymorphisms and activated microplatelets on the severity of atherosclerosis – preliminary studies
Source: Front Mol Biosci. 2026 Jan 16;12:1662954. doi: 10.3389/fmolb.2025.1662954 (PMC12856924; doi:10.3389/fmolb.2025.1662954)
Supplement: Supplementary file 1 [file Supplementaryfile1.pdf]

S1. Assessment of anthropometric, laboratory, and clinical features of atherosclerosis obliterans (AO) compared to diabetic macroangiopathy (DM)

The primary inclusion criteria for the study of patients with DM were: age 45–65 years, therapy with oral hypoglycemic agents and/or insulin, elevated glycated hemoglobin ($HbA_{1}c$) level > 6.1%, disease duration above 5 years, classification of glycemic disorders proposed by the Polish Diabetes Society (PTD) in 2023, presented in Table S1, classification of metabolic disorders, diagnostic criteria for metabolic syndrome, based on the guidelines of the World Health Organization (WHO) and the National Cholesterol Education Program (NCEP) Adult Treatment Panel III (ATP III), presented in Table 3.3. Other chronic diabetic complications: diabetic nephropathy was diagnosed based on persistent (twice measured) albuminuria (24-hour urine collection) exceeding 30mg/day in the absence of other kidney diseases, diabetic retinopathy was diagnosed based on the patients' ophthalmologic records, diabetic polyneuropathy was diagnosed based on the patients' neurological examination. The primary inclusion criteria for the study of patients with atherosclerosis obliterans were: age 40–65 years, disease duration above 5 years, clinical and biochemical signs of metabolic syndrome according to NCEP and ATP III, ankle-brachial index < 0.9 (stage II B arterial insufficiency according to Fontaine – intermittent claudication distance below 200 meters); segmental pressure measurement using Doppler ultrasound with a Sonodop 4000 device, model DSM2P, by Sonotechnik GMBH, claudication distance determined by a treadmill walking test at a speed of 3.2 km/h and a treadmill incline of 12%. The control group consisted of 18 healthy individuals, aged 44 to 65 years (mean age 57.50 ± 2.12 years), with no family history of diabetes in first-degree relatives. These individuals were free from systemic diseases, particularly: hypertension, cardiovascular diseases, lung diseases, kidney diseases, and cancer (Table S1 and Table S2).

Table S1. Classification of Glycemic Disorders According to the Polish Diabetes Association (PTD), 2023

**Measurement**	**Plasma glucose concentration**	**Interpretation**
**Random blood glucose –** measured in a blood sample taken at any time of the day, regardless of the timing of the last meal.	≥ 200 mg/dl (≥ 11.1 mmol/l)	Diagnosis of diabetes if the patient presents typical symptoms of the disease (increased thirst, polyuria, weakness, weight loss).
**Fasting blood glucose –** measured in a blood sample taken 8–14 hours after the last meal	70 – 99 mg/dl (3.9 – 5.5 mmol/l)	Normal fasting blood glucose
	100 – 125 mg/dl (5.6 – 6.9 mmol/l)	Impaired fasting glucose (IFG)
	≥ 126 mg/dl (≥ 7.0 mmol/l)	Diabetes
**Blood glucose at 120 minutes** of the oral	< 140 mg/dl (< 7.8 mmol/l)	Normal glucose tolerance

glucose tolerance test (OGTT)	140-199 mg/dl (7.8 – 11.0 mmol/l)	Impaired glucose tolerance (IGT)
	≥ 200 mg/dl (≥ 11.1 mmol/l)	Diabetes

Table S2. Criteria for the diagnosis of metabolic syndrome according to WHO and NCEP ATP III

Criteria for metabolic syndrome	According to the modified WHO criteria	According to NCEP ATP III
**Glycemia**	• Type 2 diabetes<br>• Impaired fasting glucose<br>• Impaired glucose tolerance	• fasting blood glucose ≥ 110 mg/dl
**Obesity**	• BMI >30 kg/m^2<br>• WHR male > 0.93<br>• WHR female > 0.85	• • Waist circumference men > 102 cm<br>• • Waist circumference women > 88 cm
**Hypertension**	>140/90 mmHg or use of antihypertensive medications	≥ 130/85 mmHg or use of antihypertensive medications
**Dyslipidemia**	• TG ≥ 150 mg/dl<br>• HDL male < 40 mg/dl<br>• HDL female < 35 mg/dl	• TG ≥ 150 mg/dl<br>• HDL male ≤ 40 mg/dl<br>• HDL female ≤ 50 mg/dl
**Microalbuminuria**	• microalbuminuria ≥ 20 µg/min.<br>• Albuminuria/creatinine ratio ≥ 30 mg/d	------------------------
**Diagnosed when**	Hyperglycemia + at least 2 other criteria	At least 3 criteria

Patients underwent standard laboratory tests including: 1) Biochemical tests: lipid profile, uric acid concentration, total protein, glucose; 2) Evaluation of hemostasis parameters: thrombin-antithrombin complex (TAT), von Willebrand factor (vWF), platelet-derived growth factor (PDGF), monocyte chemoattractant protein-1 (MCP-1), soluble platelet-endothelial cell adhesion molecule-1 (sPECAM-1), thrombin activatable fibrinolysis inhibitor (TAFI), 3) Evaluation of inflammation status: interleukin-6 (IL-6), fibrinogen, high-sensitivity C-reactive protein (hsCRP).

The study included 95 patients, whose general characteristics are presented in the Materials and Methods section of this work. The study group consisted of biological material (blood, urine) collected from 43 patients with AO and 34 patients with DM, while the control group consisted of material collected from 18 volunteers (Control). To conduct an initial clinical assessment of the

patients, basic anthropometric parameters were determined: age, waist-to-hip ratio (WHR), body mass index (BMI), and systolic and diastolic blood pressure. A comparative analysis was also conducted between the studied groups to check for statistically significant differences for each of the determined parameters. Detailed results of the analysis are presented in Table S3.

Table S3. Comparison of Selected Anthropometric Parameters and Biochemical Markers of DM and AO Progression Between Study and Control Groups

**Studied parametres**	**1. Patients with DM (n=34) mean ± SD**	**2. Patients with AO (n=43) mean ± SD**	**3. Control group (n=18) mean ± SD**	**p 1 v 2**	**p 1 v 3**	**p 2 v 3**
**Selected anthropometric parameters and blood pressure values.**						
**Systolic blood pressure [mmHg]**	132.32 ± 8.54	131.16 ± 9.31	128.22 ± 4.40	**0.831**	**0.245**	**0.456**
**Diastolic blood pressure [mmHg]**	80.44 ± 6.20	79.63 ± 8.03	78.33 ± 5.94	**0.881**	**0.593**	**0.808**
**BMI [kg/m^2]**	32.59 ± 6.72	27.56 ± 4.02	25.94 ± 7.92	**<0.001**	**<0.001**	**0.368**
**WHR**	0.96 ± 0.07	0.85 ± 0.05	0.80 ± 0.09	**0.193**	**<0.001**	**0.570**
**Age [years]**	60.88 ± 7.51	56.69 ± 5.72	57.50 ± 2.12	**0.388**	**0.304**	**0.640**
**Biochemical Indicators of DM and AO Progression**						
**PT [%]**	105.47 ± 8.56	103.16 ± 7.57	102.22 ± 7.67	**0.282**	**0.154**	**0.660**
**Fibrynogen [g/l]**	4.13 ± 0.54	4.41 ± 1.09	4.37 ± 1.21	**0.305**	**0.349**	**0.875**
**Total cholesterol [mg/dl]**	194.74 ± 58.09	195.93 ± 55.71	189.94 ± 51.09	**0.936**	**0.749**	**0.709**
**HDL [mg/dl]**	42.94 ± 10.75	44.31 ± 9.73	64.31 ± 8.53	**0.609**	**<0.001**	**<0.001**
**LDL [mg/dl]**	110.03 ± 40.88	124.56 ± 45.12	111.46 ± 25.36	**0.214**	**0.895**	**0.234**

**Trigliceridies [mg/dl]**	220.44 ± 250.65	141.28 ± 62.07	86.79 ± 7.25	**0.061**	**0.002**	**0.196**
**Glucose [mg/dl]**	134.94 ± 41.24	93.40 ± 10.27	94.00 ± 3.46	**<0.001**	**<0.001**	**0.930**
**Urea acid [mg/dl]**	5.91 ± 1.65	5.43 ± 1.29	5.30 ± 0.71	**0.188**	**0.118**	**0.733**
**ALAT [U/l]**	28.62 ± 29.31	26.35 ± 15.41	22.33 ± 13.40	**0.691**	**0.303**	**0.482**
**AspAT [U/l]**	26.98 ± 13.69	25.80 ± 15.92	23.42 ± 7.61	**0.752**	**0.374**	**0.526**
**GGTP [U/l]**	45.67 ± 65.23	43.57 ± 54.58	29.94 ± 22.23	**0.886**	**0.316**	**0.354**
**Urea [mg/dl]**	34.74 ± 11.42	22.80 ± 12.65	30.20 ± 9.90	**0.001**	**0.260**	**0.024**
**Creatinine [mg/dl]**	1.01 ± 0.21	1.44 ± 1.42	1.29 ± 1.16	**0.179**	**0.356**	**0.611**
**Total protein [g/dl]**	6.74 ± 1.21	6.86 ± 0.49	6.90 ± 0.54	**0.624**	**0.505**	**0.824**
**OB [mm]**	14.91 ± 15.10	12.58 ± 10.68	10.22 ± 6.04	**0.467**	**0.169**	**0.461**
**Mean Values of Biochemical Markers for Evaluating the Body's Homeostasis System**						
**vWF [%]**	174.36 ± 33.24	153.45 ± 28.12	125.89 ± 31.80	**0.013**	**<0.001**	**0.001**
**PDGF [pg/ml]**	20914.02 ± 8505.66	22806.36 ± 6494.29	16416.94 ± 7442.15	**0.346**	**0.027**	**0.002**
**TAT [ug/l]**	3.62 ± 1.18	3.34 ± 1.54	1.77 ± 0.44	**0.415**	**<0.001**	**<0.001**
**IL-6 [pg/ml]**	3.17 ± 1.53	2.80 ± 1.59	1.81 ± 0.81	**0.340**	**0.001**	**0.013**
**MCP-1 [pg/ml]**	344.45 ± 28.70	377.50 ± 152.37	282.21 ± 68.00	**0.259**	**0.035**	**0.002**
**sPECAM-1 [ng/ml]**	44.43 ± 13.72	43.19 ± 6.89	46.50 ± 4.26	**0.855**	**0.761**	**0.475**

**TAFI [%]**	108.70 ± 33.68	98.62 ± 32.15	76.59 ± 8.09	**0.168**	**<0.001**	**0.009**
**PAF-AH µmol/min/ml**	0.14 ± 0.04	0.15 ± 0.04	0.14 ± 0.02	**0.339**	**0.360**	**0.078**

A comparison of values for a randomly selected parameter (BMI) among patients with DM, AO or the control group is presented in Figure S1.

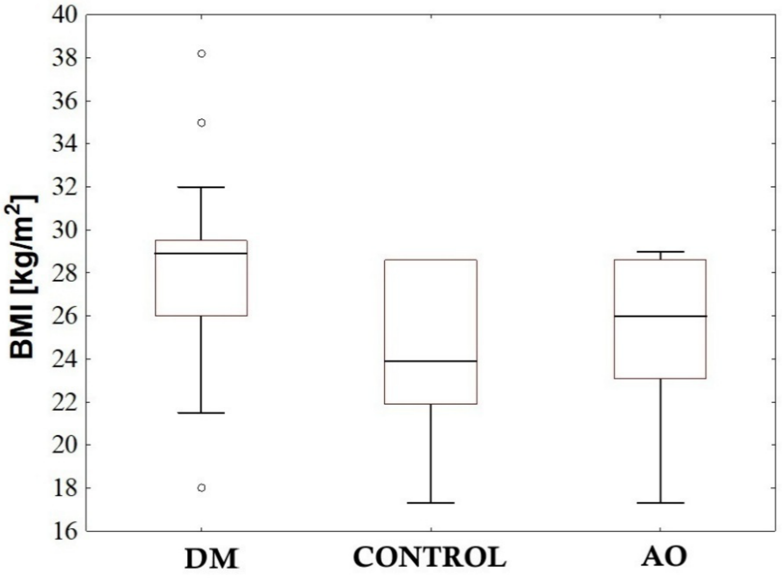

Figure S1. BMI values of patients with diabetic macroangiopathy (DM), the control group (CONTROL), and atherosclerosis obliterans (AO).

The comparison of selected anthropometric parameters among the studied patient groups and the control group revealed: Significantly lower BMI values (27.56±4.02 vs. 32.59±6.72 kg/m²; p<0.001) in patients with atherosclerosis obliterans (AO) compared to those with DM; significantly higher BMI (32.59±6.72 vs. 25.94±7.92 kg/m²; p<0.001) and WHR (0.96±0.07 vs. 0.80±0.09; p<0.001) in patients with DM compared to the control group; no significant differences in the remaining parameters analyzed when comparing the study groups with the control group. Only 6 out of 95 participants were diagnosed with hypertension (values ≥140/90 mmHg), with the reference range defined according to the guidelines of the 7th Report of the Joint National Committee on Prevention, Detection, Evaluation, and Treatment of High Blood Pressure (JNC 7).

A similar comparative analysis was conducted among patients with AO, DM and the control group for the following biochemical markers of atherosclerosis progression: fibrinogen concentration, total cholesterol, HDL cholesterol, LDL cholesterol, triglycerides, glucose, uric acid, urea, creatinine, total protein, as well as the activity of alanine aminotransferase (ALAT),

aspartate aminotransferase (AspAT), and gamma-glutamyltransferase (GGTP), prothrombin index, and erythrocyte sedimentation rate (ESR). The results are presented in Figure S2.

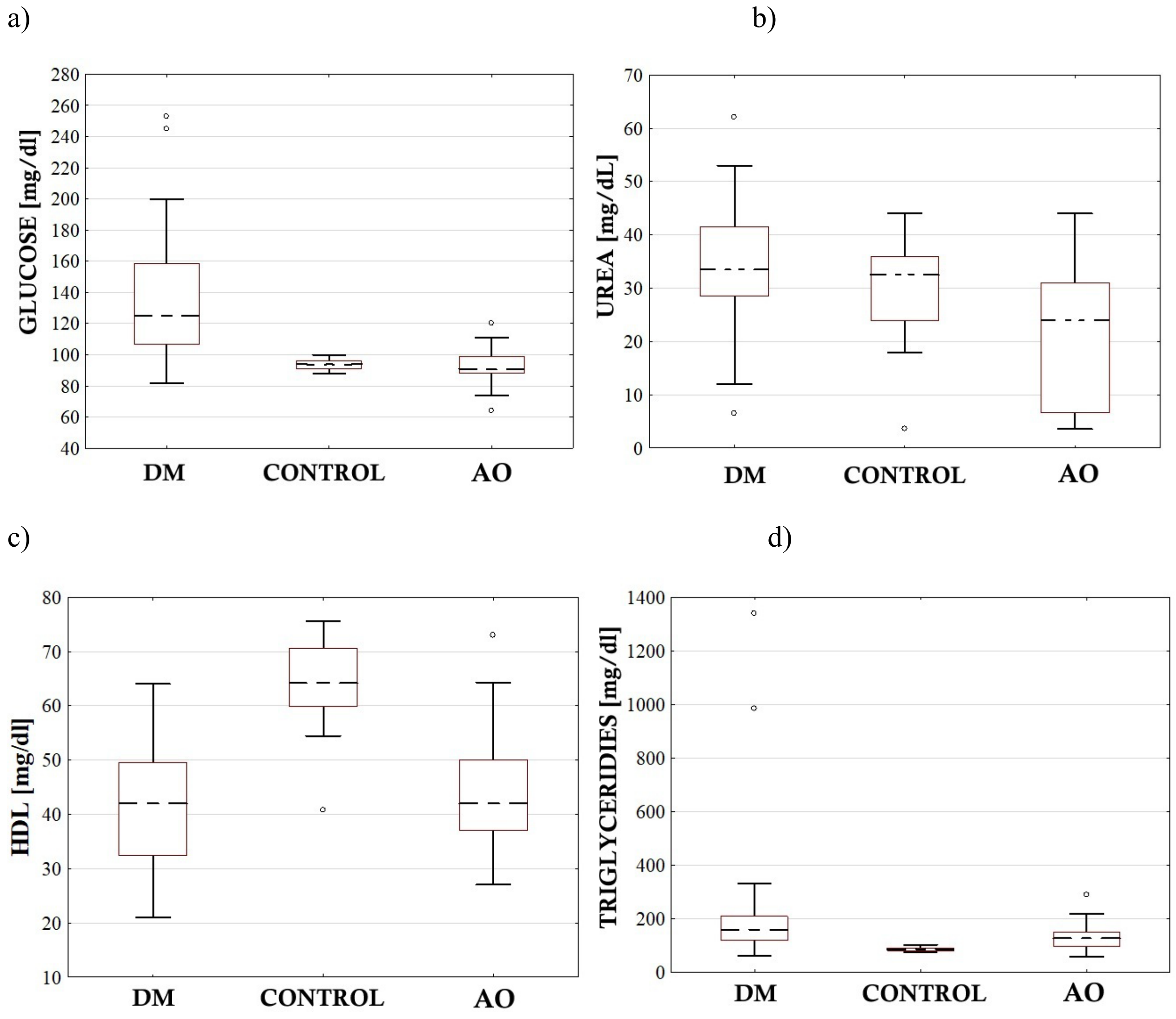

Figure S2. Values of glucose, urea, HDL, and triglyceride concentrations in blood samples from patients with diabetic macroangiopathy (DM), the control group (CONTROL), and atherosclerosis obliterans (AO).

The statistical analysis revealed: A significantly higher urea concentration (34.74±11.42 vs. 22.80±12.65 mg/dl; $p<0.001$) in the DM group compared to the AO group, indicating more frequent kidney dysfunction; A significantly lower blood glucose level (93.40±10.27 vs. 134.94±41.24 mg/dl; $p<0.001$) in the AO group compared to the DM group, which is expected due to the underlying disorder's etiology; A significant reduction in HDL fraction (44.31±9.73 vs. 64.31±8.53 mg/dl; $p<0.001$) and significantly lower urea concentration (22.80±12.65 mg/dl vs. 30.20±9.90 mg/dl; $p=0.024$) in the DM group compared to the control group; A significantly higher fasting blood glucose level (134.94±41.24 vs. 94.00±3.46 mg/dl; $p<0.001$) and an increase in triglyceride levels (220.44±250.65 vs. 86.79±7.25 mg/dl; $p<0.001$) in patients with DM compared

to the healthy control group; A significant reduction in HDL cholesterol fraction (42.94±10.75 vs. 64.31±8.53 mg/dl; p<0.001) in patients with DM compared to the control group. The degree of peripheral arterial occlusion was described by the claudication distance and ankle-brachial index value (Table S4).

Table S4. Parameters of Lower Limb Ischemia

Studied Parameters	1. Patients with DM (n=34)	2. Patients with AO (n=43)	3. Control group (n=18)	p 1 v 2	p 1 v 3	p 2 v 3
**Claudication distance [m]**	98.52 ± 73.83	147.89 ± 94.92	**----------------**	**0.014**	**----**	**----**
**Right ankle-brachial index**	0.73 ± 0.28	0.68 ± 0.23	0.75 ± 0.21	**0.413**	**0.755**	**0.290**
**Left ankle-brachial index**	0.75 ± 0.25	0.61 ± 0.29	0.69 ± 0.23	**0.070**	**0.345**	**0.327**

Based on the conducted analysis, it was found that: significantly higher claudication distances were observed in patients with AO compared to those with DM (147.89±94.92 vs. 98.52±73.83 m; p=0.014), whereas this test was not conducted in the control group and no significant differences were found between the values of the right and left ankle-brachial index. Analogous analyses were conducted for female and male groups. The analysis, considering sex, showed: significantly lower HDL levels (50.07±10.72 vs. 62.58±8.59 mg/dl; p<0.001) in female patients with DM compared to female patients with AO. Significantly lower HDL levels (40.56±9.07 vs. 50.07±10.72 mg/dl; p=0.012) in women with AO compared to women in the control group; significantly lower HDL levels (70.35±5.47 vs. 42.08±8.49 mg/dl; p<0.001) in male patients with DM compared to male patients in the control group; significantly lower HDL levels (70.35±5.47 vs. 44.83±11.80 mg/dl; p<0.001) in male patients with AO compared to male patients in the control group; significantly higher LDL levels (119.20±17.37 vs. 131.58±44.44 mg/dl; p=0.013) in women with AO compared to women with DM, which was related to the lipid-lowering therapy used by diabetic women; significantly lower uric acid levels (4.88±1.09 vs. 5.76±1.20 mg/dl; p=0.034) in women with AO compared to women with DM; significantly lower glucose levels (96.25±10.64 vs. 140.53±53.16 mg/dl; p<0.001) in women with AO compared to women with DM; significantly lower glucose levels (92.29±10.08 vs. 130.53±29.54 mg/dl; p<0.001) in men with AO compared to men with DM; significantly lower HDL levels (40.56±9.07 vs. 62.58±8.59 mg/dl; p<0.001) in women with DM compared to women in the control group; significantly higher glucose levels (140.53±53.16 vs. 94.07±3.05 mg/dl; p<0.001) in women with DM compared to women in the control group; significantly higher glucose levels (130.53±29.54 vs. 93.75±3.86 mg/dl; p<0.001) in men with DM compared to men in the control group.

Anthropometric parameters assessed in clinical diagnostics include height, body weight, BMI, WHR, chest circumference, percentage of body fat, and percentage of muscle mass. It is well known that both overweight and obesity (measured by BMI and body weight) are strongly associated with the risk of atherosclerosis. Excess fat puts strain on the heart and can lead to cardiovascular diseases, as visceral fat promotes inflammation and metabolic disorders. Similarly, central obesity, measured by waist circumference and WHR, is closely linked to atherosclerosis risk. Abdominal fat is metabolically active, can induce inflammation, and contributes to insulin

resistance, which frequently leads to T2DM and, consequently, the development of DM (Friedman et al., 2016). On the other hand, low muscle mass may also increase the risk of atherosclerosis, as muscles play a key role in glucose and fat metabolism.

The study demonstrated significant differences in BMI and WHR values in patients with vascular complications compared to healthy individuals of similar age and the same ethnic population. Both parameters were significantly higher in individuals with AO and DM compared to the control group. These findings align with results from other research groups, which have shown that the progression of atherosclerosis is related to BMI. It has been demonstrated that every one-unit increase in BMI above the normal range increases the risk of atherosclerosis and coronary artery disease by 10%, while a 10 kg weight gain raises the risk by 12% (Friedman et al., 2016). Additionally, arterial stiffness, which stimulates excessive collagen production and deposition in tissues leading to atherosclerosis progression, increases in patients with severe obesity (BMI ≥ 40 kg/m²) (Gosk-Bierska et al., 2016). Inflammation in blood vessels caused by obesity is driven by adipokine and cytokine activation and increased aldosterone levels in the blood. Adipokines and cytokines, such as IL-1β, IL-18, and TNF, contribute to endothelial dysfunction and increase endothelial permeability. Elevated aldosterone levels increase blood volume, promote platelet aggregation, endothelial dysfunction, thrombosis, and fibrosis. Moreover, adipokines such as leptin, resistin, IL-6, and monocyte chemoattractant protein-1 (MCP-1) attract monocytes/macrophages to adipose tissue (where macrophages can constitute up to 40% of all fat tissue cells in obesity (Hagag et al., 2014)), inducing visceral fat inflammation, systemic inflammation, oxidative stress, lipid oxidation, insulin resistance, endothelial dysfunction, and hypercoagulability—all of which contribute to atherosclerosis progression (Friedman et al., 2016). Obesity also accelerates the clinical onset of insulin resistance and T2DM, which further induce and sustain chronic inflammation, leading to DM (Hagag et al., 2014). In obesity, macrophages polarize into pro-inflammatory M1 macrophages, which secrete pro-inflammatory cytokines, disrupting insulin signaling and inducing insulin resistance. Additionally, high BMI in type 1 diabetes (T1DM) accelerates the progression of both macro- and microangiopathy(Hamilton and Trejo, 2017). This study demonstrated that BMI was significantly higher in the DM group compared to both the AO group and the control group. A similar trend was observed for WHR. Given that two billion adults worldwide (30% of the global population) are overweight or obese, contributing to over 3.4 million deaths annually, and considering that obesity is an independent risk factor for atherosclerosis, it is crucial to implement effective strategies to reduce BMI and WHR in obese patients. The relationship between obesity, adipose tissue inflammation, and metabolic diseases suggests that targeting inflammatory pathways could be a promising therapeutic approach for metabolic complications, including DM, AO, and other obesity-related pathologies (Hagag et al., 2014).

A key role among the metabolic disorders characteristic of diabetes that contribute to the progression of atherosclerosis is played by prolonged exposure to hyperglycemia and insulin resistance, especially when combined with risk factors such as obesity, hypertension, and dyslipidemia. Laboratory and clinical studies conducted in numerous research centers unanimously confirm that the development of DM is caused by the overproduction of reactive oxygen species (ROS), oxidative stress, excessive formation and activation of various advanced glycation end-products (AGEs), AGE-RAGE interactions, polyol and hexosamine pathway activation, and protein kinase C activation. These processes lead to chronic vascular inflammation (Borissoff et al., 2010). It is now recognized that persistent hyperglycemia is the primary cause of vasculopathy in T2DM —hyperglycemia stimulates apoptosis, reduces endothelial cell (EC) replication, and accelerates atherosclerosis progression (Hirano, 2007). Furthermore, insulin resistance in

hepatocytes and myocytes is not only the main cause of the onset and progression of diabetes but also a significant risk factor for the development and progression of other atherosclerotic diseases, such as hypertension and dyslipidemia (Hung et al., 1992). The most significant factors contributing to the development of atherosclerosis in diabetes include: duration of diabetes, HbA_{1c} concentration, presence of arterial medial calcifications. These factors increase the risk of death from coronary artery disease up to threefold compared to individuals without carbohydrate metabolism disorders (Cassar et al., 2005). Therefore, an obvious parameter differentiating patients with DM from those with AO or healthy individuals is glucose concentration. In the present study, significantly lower glycemia was observed in the AO patient group compared to the DM patient group, while glucose levels were significantly higher in the DM group compared to the control group. A comparative analysis of glycemic values by sex also showed that both female and male patients with AO had significantly lower glucose levels compared to those with DM. Similarly, female patients with DM had significantly higher glucose levels than women in the control group, and the same pattern was observed among men. Due to the lack of HbA_{1c} data, it was not possible to determine its role in differentiating AO from DM or DM from the control group. However, significantly lower urea levels were found in the AO patient group compared to the DM group, likely due to impaired kidney function in DM. The association between elevated urea levels and vascular disorders has already been observed in previous studies (Iso et al., 2010; Jakobsche-Policht et al., 2014).

In the presented study, a significant reduction in HDL levels was observed in patients with AO and those with DM compared to the control group. This relationship is commonly observed in other scientific studies (Jennings, 2009). Additionally, the DM group showed significantly higher triglyceride levels than the control group, which is also a frequently observed trend (Katakami, 2018; Li et al., 2020). It should also be clarified that the significantly higher LDL levels in women with AO compared to those with DM were due to the lipid-lowering therapy administered to the diabetic women. Furthermore, in diabetes, an increase in uric acid levels is often observed compared to individuals without carbohydrate metabolism disorders. It has been shown that in T2DM, uric acid levels are significantly associated with microalbuminuria, even in patients with normal serum creatinine levels, which could be important in the early detection of diabetic nephropathy. Uric acid levels may therefore serve as a potential screening tool for the early detection of diabetic kidney disease (Libby and Theroux, 2005).

S2. Evaluation of microparticles (PMPs) and platelets (PLT) aggregation

Methods: Optical aggregometry based on visible light measurement (Light Transmission Aggregometry, LTA) is considered the gold standard for diagnosing platelet function disorders. A platelet aggregation agonist, such as TRAP, ADP, or collagen, is added to a sample of platelet-rich plasma (PRP) at an appropriate concentration, initiating platelet aggregate formation. Interaction between platelets and the agonist leads to platelet activation and subsequent aggregation. The formation of platelet aggregates is accompanied by a proportional increase in light transmittance through the measurement cuvettes. To determine platelet aggregation capacity in each plasma sample (containing isolated platelets), a turbidity measurement method was used. Platelet aggregation in these samples was induced by an initiating factor—TRAP, ADP, or collagen. Upon addition of the factor, platelet clusters (aggregates) formed, increasing light transmittance through the plasma. Continuous measurement of light passing through the plasma allowed for the generation of an aggregation curve, representing platelet responsiveness to activators. High

aggregation threshold values for a given factor indicated increased platelet aggregation susceptibility, suggesting platelet activation in the patient. Reduced aggregation levels could result from the use of nonsteroidal anti-inflammatory drugs (NSAIDs) or platelet dysfunction (either congenital or acquired). In the first stage, platelet concentration in plasma (previously collected into sodium citrate tubes) was adjusted to 250,000 platelets in 250 µL by diluting platelet-rich plasma (PRP) with autologous platelet-poor plasma (PPP). A control sample contained 250 µL of PPP. Subsequently, 25 µL of the aggregation factor was introduced into the stabilized experimental system at a previously determined concentration (ADP, TRAP, or collagen). Aggregation measurement was conducted for 7 minutes. Centrifugation procedures were performed using an MPW-360 centrifuge with a horizontal rotor (IKA, Poland). Quantitative assessments were performed under a light microscope (Jenomed, Carl Zeiss, Germany). Platelet aggregation was evaluated using an APACT aggregometer (LAbor, Hamburg, Germany). Determining platelet aggregation type in vitro is an attempt to characterize the ability of platelets to form the primary hemostatic plug in vivo. Aggregation induced by collagen (Cat. 385, Chrono-Log, Havertown, PA, USA) at a concentration of 5 µg/mL is used to assess overall platelet aggregation capacity. A delay phase of up to 1 minute is a typical phenomenon with this agonist, leading to ATP secretion preceding platelet aggregation. Collagen activates platelet aggregation through the glycoprotein VI (GP VI) receptor on the platelet surface (reference aggregation values: 70-100%). Adenosine diphosphate (ADP) is a natural platelet aggregation agonist that activates platelets by interacting with specific purinergic receptors—P2Y1 and P2Y12. Platelet stimulation with ADP (Cat. 384, Chrono-Log, Havertown, PA, USA) at a concentration of 5–20 µM initially leads to an increase in intracellular calcium (Ca^{2+}) levels, accompanied by inhibition of adenylate cyclase (AC). As a result, synthesis of cyclic adenosine monophosphate (cAMP), an inhibitor of the aggregation process, does not occur. The intracellular Ca^{2+} increase induced by ADP stimulation is due to the activation of membrane calcium channels, primarily the P2X1 purinergic receptor, which allows calcium influx into platelet cytoplasm. Since ADP is stored in platelet dense granules, platelet activation leads to granule content release (including ADP), which enhances aggregation and results in the second phase of the aggregation curve. Impairment of platelet activation via the ADP pathway leads to an abnormal response to other agonists (normal aggregation range: 70-90%). Platelet aggregation induced by thrombin receptor-activating peptide (TRAP) (Thrombin Receptor Activator Peptide 6, Sigma-Aldrich, Cat. T1573, St. Louis, MO, USA) at a concentration of 10 µM/200 µL of the test material (250,000 platelets/200 µL PRP) is measured by percentage change in sample turbidity and the reaction time. TRAP induces platelet activation, adhesion, and aggregation through the thromboxane (TXA2) platelet receptor (normal aggregation range: 80-100%). The image of microparticles in the sample was assessed using flow cytometry. The cellular material was appropriately stained to visualize the adhesive integrin receptor GP3A and β3 (CD61). The percentage of target units was measured after setting the appropriate density and cell size gating, based on literature data defining the location of the analyzed microparticles within the sample. The values were expressed as the number of stained structures evaluated per 10,000 events.

Results:

Determining the type of PLT aggregation in biological material helps assess the predisposition of PLT to form the primary hemostatic plug in vivo. PLT can be activated by various hemostatic factors, including collagen (which activates PLT aggregation through the main platelet receptor glycoprotein VI (GP VI)), adenosine diphosphate (ADP) (which activates PLT via purinergic receptors P2Y1 and P2Y12), and thrombin (which activates PLT via the thromboxane receptor, TXA2). In this study, an attempt was made to determine the levels of both inactive and activated

PLT in the plasma of AO patients compared to DM patients and the control group. Additionally, an original approach was taken to investigate the significance of activated PMPs in the studied groups. PLT activation was induced using collagen, ADP, and thrombin receptor-activating peptide (TRAP, a thrombin analogue), while PMP activation was assessed using TRAP alone. The obtained results are presented in Figure S3.

A)

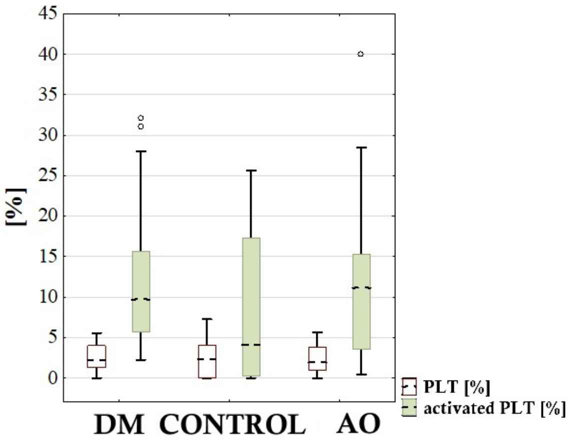

B)

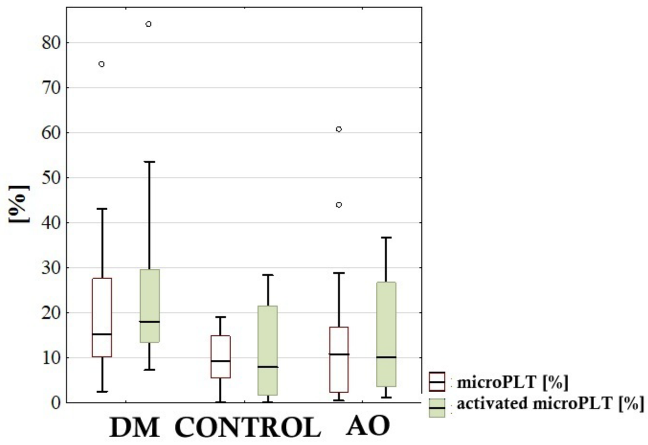

Figure S3. Percentage distribution of: A) platelets; B) microparticles before and after TRAP activation in patient groups with diabetic macroangiopathy (DM), control (CONTROL), and atherosclerosis obliterans (AO).

Based on the obtained results, significant differences were demonstrated in the percentage shares of PMP activated by TRAP among patients with AO, DM and the control group. It was shown that: The level of activated PMP was significantly lower in patients with AO compared to patients with DM (15.56±13.82 vs. 23.25±15.95%; p=0.045); a significant increase in both non-activated (19.21±14.33 vs. 9.59±5.80%; p=0.007) and activated microplatelets (23.25±15.95 vs. 11.51±10.70%; p=0.003) was observed in patients with DM compared to the control group. However, no statistically significant differences were found between: the level of activated PMP in the AO group and the control group; the level of PLT in the studied groups (AO, DM and control) before and after activation with collagen, ADP, or TRAP; additionally, an analysis considering gender was conducted, which revealed: A significant increase in both non-activated (19.5±10.15 vs. 9.01±6.01%; p=0.021) and activated microparticles (25.57±11.13 vs. 11.11±10.40%; p=0.002) in women with DM compared to women in the control group; a significant increase in non-activated (16.00±7.82 vs. 9.01±6.01%; p=0.022), activated microparticles (22.82±10.55 vs. 11.11±10.40%; p=0.012), and activated platelet aggregates (15.90±8.44 vs. 8.42±8.97%; p=0.038) in women with AO compared to women in the control group; a significant increase in activated platelet aggregates (2.79±1.29 vs. 1.04±1.98%; p=0.038) in men with DM compared to men in the control group. No significant differences were found in the levels of activated PMP, non-activated PMP, activated platelet aggregates, or non-activated platelet aggregates between women with AO and women with DM, nor between men with AO and men with DM. The statistical analysis considering gender within a given disease revealed significant differences in the levels of activated and non-activated

PLT and PMP only among patients with AO. It was demonstrated that women with AO had significantly higher levels of activated PMP (22.22±11.38 vs. 12.98±13.98%; p=0.036) and activated platelet aggregates (16.74±11.32 vs. 8.89±6.13%; p=0.003) compared to men with AO. The level of activated platelet aggregates was significantly higher in women with AO compared to all AO patients (16.74±11.32 vs. 11.13±8.59%; p=0.029). No significant differences were observed in the levels of activated and non-activated PLT and PMP in analyses considering gender and disease depending on the activating factor used, i.e., collagen, ADP, or TRAP.

Additionally, imaging of the adhesive receptor – integrin GP3A and β3 (CD61) on the surface of activated and non-activated PLT was performed. Together with CD41, it forms the glycoprotein IIb-IIIa complex, a crucial mediator of platelet aggregation. The quantitative assessment of PMP in the PLT population is presented in Figure S4.

A) B)

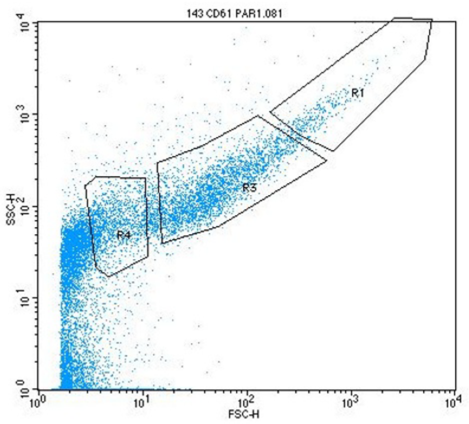

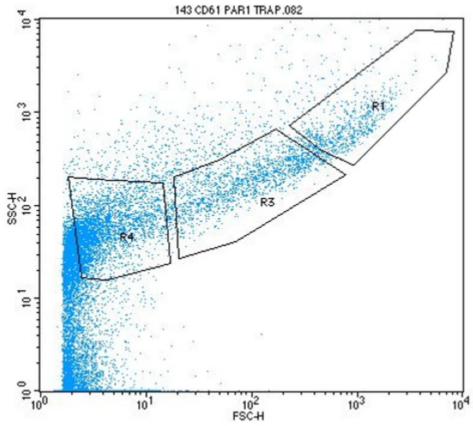

Figure S4. Flow cytometric analysis of PAR-1 expression in human resting platelets (A) and platelets activated with 10 μM TRAP (B). Platelets were gated based on the presence of CD61-FITC antigen and labeled with antibodies against PAR-1-APC antigen. The designated regions P4, P3, and P1 correspond to microparticles, normoplatelets, and platelet aggregates, respectively. Image of a patient with DM.

Flow cytometric analysis included the preliminary identification and localization of platelets based on FSC (Forward Scatter), differentiating structures by size, and SSC (Side Scatter), differentiating structures by the density of internal granules. To achieve this, the R2 gate was set in the statistical analyses of the FACS Canto BD cytometer software, and the number of recorded events was automatically counted in a flow of 10,000 cellular objects. Next, the fluorescence intensity of PAR-1 labeled with APC on the surface of CD61-FITC-labeled platelets was analyzed, with the P1 gate being set for this purpose. To determine the appropriate platelet population, markers M1 and M2 were analyzed for the examined structures. Data analysis was performed for individual patients in two study models: in isolated platelets before and after TRAP activation. The presented images suggest a significant increase in the number of labeled PAR-1 receptors on the platelet surface after TRAP stimulation. The observed increase in PAR-1 receptor activation was particularly evident in patients with DM.

Moreover, activated PLT release various chemokines, such as platelet factor 4 (PF4), growth factors, and serotonin, which stimulate VSMC proliferation. PLT are also a source of proteins involved in the coagulation system, including factors V, XI, and XIII, as well as proteins related to fibrinolysis, such as plasminogen and plasminogen activator inhibitor-1 (PAI-1). Fibrinogen, fibrin, and its degradation products play crucial roles in the early stages of atherosclerotic changes, with their levels increasing as intimal thickening progresses and plaque develops (Bini et al., 1989). Fibrin degradation products stimulate monocyte migration and IL-6 production. The inflammation developing within the arterial wall, supported by coagulation-related processes, can enhance local thrombin production, inducing a self-sustaining cycle of pathophysiological events. This contributes to increased thrombus formation within atherosclerotic plaques, ultimately leading to their destabilization (Kramer et al., 2010). As a result, cytokine levels, including IL-6, rise, with IL-6 being released by macrophages. One of its primary functions is to regulate immune responses by influencing B-cell activation and proliferation. IL-6 also stimulates the liver to synthesize acute-phase proteins such as fibrinogen, CRP, and serum amyloid A, thereby linking the local inflammatory response with a systemic reaction. The findings of this study also demonstrated that IL-6 is a distinguishing parameter between patients with atherosclerotic disorders (DM or AO) and healthy individuals. A gender-based analysis further revealed that both women and men with DM exhibited significantly higher plasma IL-6 levels than their respective healthy counterparts. Similar observations were made by another research team, which not only found significantly elevated IL-6 levels in patients with DM compared to healthy volunteers but also demonstrated that IL-6 levels correlated with arterial calcification (Saremi et al., 2009). The ongoing inflammatory state is associated with an intensified process of neovascularization. Although this phenomenon is part of the repair mechanisms triggered by vascular wall damage and facilitates the formation of collateral circulation, it also promotes the progression of atherosclerosis. It has been observed that plaques prone to rupture exhibit a twofold increase in the number of microvessels, while in ruptured plaques, this increase can be up to fourfold. In clinical practice, the number of microvessels is considered to correlate with disease severity. Microvessels formed within atherosclerotic plaques exhibit a distinct branching structure and composition compared to those in the adventitia. The invasion of the vasa vasorum (a network of microvessels within the outer layers of arteries and veins that supply large blood vessels) occurs mainly in areas of medial layer rupture, particularly near the necrotic core. These vessels form numerous branches around the external surface of the core. Most microvessels in atherosclerotic lesions remain immature, characterized by smaller diameters than those in the adventitia and consisting mainly of endothelial cells with few pericytes and VSMCs. They are prone to rupture, which can lead to intraplaque hemorrhage and microthrombus formation. The activation of coagulation processes stimulates VSMC proliferation and promotes their production of connective tissue components. As a result, seemingly asymptomatic microcirculatory hemorrhages can lead to the rapid progression of atherosclerosis. Neovascularization thus plays a key role in plaque rupture. Additionally, microvessels exhibit increased permeability to blood components, facilitating the migration of inflammatory cells from the bloodstream into the vessel wall. This promotes macrophage infiltration of the plaque, further driving angiogenesis. A positive feedback loop exists: newly formed vessels allow inflammatory cells to enter the vessel wall, which in turn stimulates further blood vessel formation. Studies have shown that the number of microvessels correlates with the number of inflammatory cells present in atherosclerotic lesions rather than with plaque thickness itself. The angiogenesis process in atherosclerosis is complex, involving extracellular matrix autolysis, EC, VSMC, and pericyte migration and proliferation, as well as the formation and maturation of new vascular structures (Jaipersad et al., 2014). The main factors

stimulating angiogenesis are hypoxia and local inflammatory processes, which activate the expression of growth factors such as vascular endothelial growth factor (VEGF), platelet-derived growth factor (PDGF), and basic fibroblast growth factor (b-FGF), along with their receptors. VEGF stimulates endothelial cell proliferation and initiates angiogenesis, while PDGF and b-FGF are responsible for the maturation of newly formed vascular structures. PDGF is considered a key factor in the maturation of new vessels (Boyle et al., 2017), regulating and differentiating cell divisions, thereby influencing vascular tone. PDGF and other growth factors stimulate smooth muscle cells in the medial layer to migrate toward the upper layers of the intima, where they proliferate and produce connective tissue components such as collagen, elastin, and proteoglycans. This process leads to the formation of a fibrous cap covering the primary atherosclerotic lesion. The formation of this connective tissue cap aims to shield toxic elements within the atherosclerotic plaque and is considered a sign of beneficial healing processes. Additionally, PDGF is regarded (similar to PF-4) as a marker of PLT activity. The present study demonstrated significantly elevated PDGF levels in patients with DM and OA compared to healthy volunteers.

PMPs and endothelial-derived microparticles (EMP) play a crucial role in angiogenesis (Mezentsev et al., 2005), as they serve as carriers of intercellular signals. They participate in all stages of angiogenesis, including proliferation, migration, and the formation of vessel-like structures, by stimulating endothelial cell movement and the formation of new blood vessels (Morel et al., 2004). In conditions of endothelial dysfunction and inflammatory states such as coronary artery disease or thrombotic microangiopathies, a significant increase in the concentration of PMP, EMP, and monocytes has been observed. In vascular diseases, changes in the composition of microparticles can be detected. Circulating microparticles reflect cellular activation processes and damage to both circulating and stationary vascular system cells (Kailashiya, 2018). EMPs express adhesion molecules, with the most characteristic antigen combinations being CD105+/CD144+. An increase in the population of microparticles exhibiting this antigen set may indicate vascular damage, which in turn increases the risk of thrombotic complications. EMPs also express vWF multimers (Simak and Gelderman, 2006).

In patients with varying degrees of coronary artery disease, an increase in EMP (CD31+, CD42-) has been observed (Tan et al., 2005b). A direct correlation has been demonstrated between the number of these microparticles and angiographic vascular imaging, especially in cases of left anterior descending artery stenosis compared to the number of microparticles in patients with right coronary artery stenosis. Furthermore, an increase in the number of EMPs (CD31+, annexin V+) correlates with endothelial dysfunction, which can be assessed angiographically by a decrease in vascular tone in response to acetylcholine administration (Werner et al., 2006). An increase in PMP levels is primarily found in cases of cerebrovascular occlusion, which may indicate platelet activation in small vessels (Burnouf et al., 2014). In stable coronary artery disease, an increase in PMP (CD61+, CD42b+) has been observed, which may signal a prothrombotic state. In patients with abdominal aortic aneurysms, active TF was observed on the surface of PMP, suggesting interactions between platelets and monocyte-derived microparticles and their involvement in thrombus formation (Touat et al., 2006). An increase in the subpopulation of PMP marked as CD63+ and P-selectin+ in individuals with PAD and myocardial infarction correlates with the early phase of myocardial infarction and arterial atherosclerosis (Van Der Zee et al., 2006). High levels of PMP marked as CD62+ and CD63+ are observed in patients with intermittent claudication and those who have experienced ischemic events (Tan et al., 2005a).

Based on the results obtained and presented in this study, significant differences were demonstrated in the percentage distribution of activated PMP in the groups of patients with AO,

DM and the control group. A significantly lower level of activated PMP was observed in patients with AO compared to patients with DM, while there was a significant increase in both non-activated and activated PMP levels in patients with DM compared to the control group. Gender-based analysis revealed a significant increase in the levels of both non-activated and activated PMP in the women with DM compared to women in the control group, a significant increase in non-activated and activated PMP levels, as well as activated platelet aggregates in the women with AO compared to women in the control group, and a significant increase in the level of activated platelet aggregates in the men with DM compared to men in the control group. Statistical analysis considering gender within a given condition demonstrated a significantly higher level of activated PMP and activated platelet aggregates in women with AO compared to men with AO and to the entire patient group with AO.

Based on the conducted research, it can be concluded that PMP are a probable marker for differentiating patients with DM from those with AO. The obtained results are consistent with observations from other research teams, which also reported an increase in the number of circulating PMP in various systemic diseases, especially in diabetes. This suggests activation of the cells from which they originate and the involvement of microparticles in endothelial damage (Omoto et al., 2002). In T1DM, an elevated level of PMP, EMP, and microparticles with exposed phosphatidylserine, as well as an increase in the procoagulant activity of these microparticles, is observed, which correlates with $HbA_{1}c$ levels (Sabatier et al., 2002). This suggests a link with carbohydrate metabolism. In T2DM, only a significantly higher total number of microparticles with exposed phosphatidylserine was observed, but their procoagulant activity was not demonstrated. Additionally, no increase in the number of PMP (CD42+ and CD41a+) or monocytic microparticles marked as CD14+ was observed. The lack of an observed relationship between PMP in T2DM is most likely due to the fact that the studied group of patients did not show complications characteristic of macroangiopathy (possibly because the duration of diabetes was short). However, there is no information in the current literature indicating that any research team has examined the relationship between the increase in PMP numbers and the degree of progression of DM.

It is well known that patients with T2DM exhibit high levels of activated PLT (Mahmoodian et al., 2019), which, at sites of vascular wall damage, cause interactions between numerous platelet glycoprotein receptors and collagen fibers (Sachs et al., 2022). However, an elevated concentration of activated PMP in T2DM has not yet been demonstrated. The observation presented in this study, indicating significantly higher levels of activated PMP in patients with DM compared to those with AO, is a pioneering finding that requires confirmation through similar experiments conducted on a significantly larger group of patients with AO and DM. The obtained results might then potentially allow for a definitive assessment of the significance of activated PMP as a reliable marker distinguishing DM and AO.

S3. Quantitative Assessment of Selected Hemostasis and Inflammation Parameters

Methods:

The quantitative assessment of von Willebrand factor (vWF) in plasma samples from patients was performed using the enzyme immunoassay (EIA) test (Asserachrom vWF, no. cat. 11875396, Diagnostica Stago, Asnières-sur-Seine, France) for diagnostic purposes (reference range: 50-160%). To prepare the sample for the experiment, 1 part of the patient's blood plasma, collected in a tube with 3.2% sodium citrate, was mixed with 9 parts of the patient's venous blood. The mixture was immediately centrifuged (MPW-310, IKA, Poland) at 2500 x g at room temperature for 15

minutes and stored at -40°C. Before the experiment, the samples were thawed by 10-minute incubation at 37°C and then diluted 51 times. The EIA test was conducted according to the manufacturer's guidelines, following the recommended incubation and washing conditions (no. cat. 11875396). Absorbance values were measured photometrically (Multiscan FC, Thermo Fisher Scientific, Massachusetts, GA, USA) at a wavelength of 492 nm.

Quantitative evaluation of the thrombin-antithrombin III (TAT) complex in plasma samples was conducted using the ELISA immunoenzymatic test (Enzygnost TAT micro, no. cat. OWMG G15E4141 S/CS, Dade Behring, Frankfurt, Germany). To prepare the sample for the experiment, 1 part of the patient's plasma (collected in a tube with 3.2% sodium citrate) was mixed with 9 parts of the patient's venous blood, immediately centrifuged (MPW-310, IKA, Poland) at 1500 x g for 15 minutes at room temperature, and stored at -40°C. The ELISA test was performed according to the manufacturer's guidelines, adhering to the incubation and washing conditions (OWMG G15E4141 S/CS). Absorbance values were measured photometrically (Multiscan FC Thermo Fisher Scientific, Massachusetts, GA, USA) at 492 nm.

The quantitative evaluation of isoform AB of platelet-derived growth factor (PDGF) in plasma samples from patients was conducted using ELISA plates (Quantikine® Human PDGF-AB, no. cat. DHD00B, R&D Systems, Minneapolis, Canada) for diagnostic research purposes (reference range: 1.0–4.1 µg/l). The plasma samples were collected in EDTA tubes, placed on ice, immediately centrifuged (MPW-310, IKA, Poland) at 2–8°C and 1000 x g for 15 minutes, and stored at -40°C. The samples were thawed and diluted 50 times with Calibrator Diluent RD6-11 buffer before conducting the ELISA test according to the manufacturer's guidelines. Absorbance values were measured photometrically (Multiscan FC Thermo Fisher Scientific, Massachusetts, GA, USA) at 450 nm.

The quantitative assessment of monocyte chemoattractant protein-1 (MCP-1) in serum samples from patients was performed using the ELISA test (Human MCP-1, no. cat. BMS281CE, Bender MedSystems, Vienna, Austria), designated for scientific research (reference range: not available). Blood samples were collected in serum separator tubes, allowed to clot, and then centrifuged (MPW-310, IKA, Poland) at room temperature at 700 x g for 10 minutes and stored at -40°C. Prior to testing, the samples were thawed and diluted 5 times with assay buffer. The ELISA test was conducted according to the manufacturer's instructions, using plates coated with anti-MCP-1 monoclonal antibodies and following the incubation and washing conditions (no. cat. BMS281CE). Absorbance was measured photometrically (Multiscan FC Thermo Fisher Scientific, Massachusetts, GA, USA) at 450 nm.

The quantitative assessment of interleukin 6 (IL-6) in serum samples from patients was carried out using the ELISA test (Human IL-6, no. cat. BMS213/2CE, Bender MedSystems, Vienna, Austria), designated for scientific research (reference range: not available). Serum was collected in serum separator tubes, allowed to clot, centrifuged (MPW-310, IKA, Poland) at room temperature at 700 x g for 10 minutes, and stored at -40°C. Prior to analysis, the samples were thawed and diluted 2 times with assay buffer. The ELISA test was conducted according to the manufacturer's instructions, using anti-IL-6 monoclonal antibody-coated plates and observing the specified incubation and washing conditions (no. cat. BMS213/2CE). Absorbance was measured photometrically (Multiscan FC Thermo Fisher Scientific, Massachusetts, GA, USA) at 450 nm.

The quantitative evaluation of soluble platelet-endothelial cell adhesion molecule-1 (sPECAM-1) in serum samples was performed using the ELISA test (Human sPECAM-1, no. cat.

BMS229, Bender MedSystems, Vienna, Austria), designated for scientific research purposes (reference range: not available). Serum was collected in serum separator tubes, allowed to clot, centrifuged (MPW-310, IKA, Poland) at room temperature at 700 x g for 10 minutes, and stored at -40°C. Before testing, the samples were thawed and diluted 10 times with the sample diluent. The ELISA test was conducted following the manufacturer's guidelines on plates coated with anti-sPECAM-1 monoclonal antibodies. Absorbance values were measured photometrically (Multiscan FC Thermo Fisher Scientific, Massachusetts, GA, USA) at 450 nm.

The quantitative assessment of platelet-activating factor acetylhydrolase (PAF-AH) in serum samples was carried out using the ELISA test (PAF Acetylhydrolase Assay Kit, no. cat. 760901, Cayman Chemical Company, Michigan, USA), designated for scientific research purposes (reference range: not available). To prepare the sample, 1 part of the patient's plasma, collected in a tube with 3.2% sodium citrate, was mixed with 9 parts of venous blood, immediately centrifuged (MPW-310, IKA, Poland) at 1500 x g for 15 minutes at room temperature, and stored at -40°C. Before testing, the samples were thawed and subjected to molecular filtration using 30 kDa cutoff columns (Amicon® Ultra Centrifugal Filter, Sigma-Aldrich, St. Louis, MO, USA) to concentrate the enzyme. The enzyme activity was measured by adding 10 µl of control (non-enzymatic), positive control, and sample to the ELISA plate, followed by 10 µl of DTNB (5,5'-dithiobis-2-nitrobenzoic acid) and 5 µl of assay buffer. The reaction was initiated by adding 200 µl of substrate solution and mixing. Absorbance was measured at 405–414 nm at 5 time points, every 60 seconds.

Quantitative assessment of the thrombin-activated fibrinolysis inhibitor (TAFI) in plasma samples was performed using the ELISA test (IMUCLONE® TAFI ELISA, no. cat. 873, American Diagnostica Inc., NY, USA) for scientific research purposes (reference range: not available). The preparation of the sample followed the same procedure as described for PAF-AH, with plasma mixed with 3.2% sodium citrate and venous blood. After centrifugation, the plasma was stored at -40°C. Prior to testing, the samples were thawed and diluted 50 times using Sample Diluent-F solution. The ELISA test was performed according to the manufacturer's instructions, observing incubation and washing conditions (no. cat. 873). Absorbance values were measured photometrically (Multiscan FC Thermo Fisher Scientific, Massachusetts, GA, USA) at 450 nm.

During hospitalization, the following laboratory diagnostic parameters were routinely assessed: hematological profile (5-diff; 16-parameter hematology analyzer ABX MICROS OT, Horiba Medical, Japan, reagents by ABX Roche Diagnostic Systems, Switzerland), nitrogenous metabolites (urea, creatinine), lipid profile (total cholesterol, LDL and HDL fractions, triglycerides), inflammatory markers (hsCRP, ESR), fibrinogen, uric acid, total protein, glucose, coagulation parameters (APTT, prothrombin time, INR), claudication distance and ankle-brachial index. Diagnostic tests were performed at the Diagnostic Laboratory of the University Hospital in Wroclaw.

Results:

In the first stage of the research, a quantitative assessment of the following parameters was conducted: von Willebrand factor (vWF), thrombin-antithrombin III complex (TAT), interleukin 6 (IL-6), platelet-derived growth factor (PDGF), monocyte chemoattractant protein 1 (MCP-1), soluble platelet-endothelial cell adhesion molecule-1 (sPECAM-1), platelet-activating factor acetylhydrolase (PAF-AH), and thrombin-activatable fibrinolysis inhibitor (TAFI). These factors were selected to indicate changes occurring at different stages of the atherosclerosis pathomechanism, including platelet activation (TAT, TAFI), platelet adhesion to the endothelium (vWF), development of inflammation (IL-6), induction of foam cell formation (MCP-1

chemokine), and plaque rupture associated with neovascularization (PDGF). The results of the quantitative analysis of selected factors are presented in Figure S5.

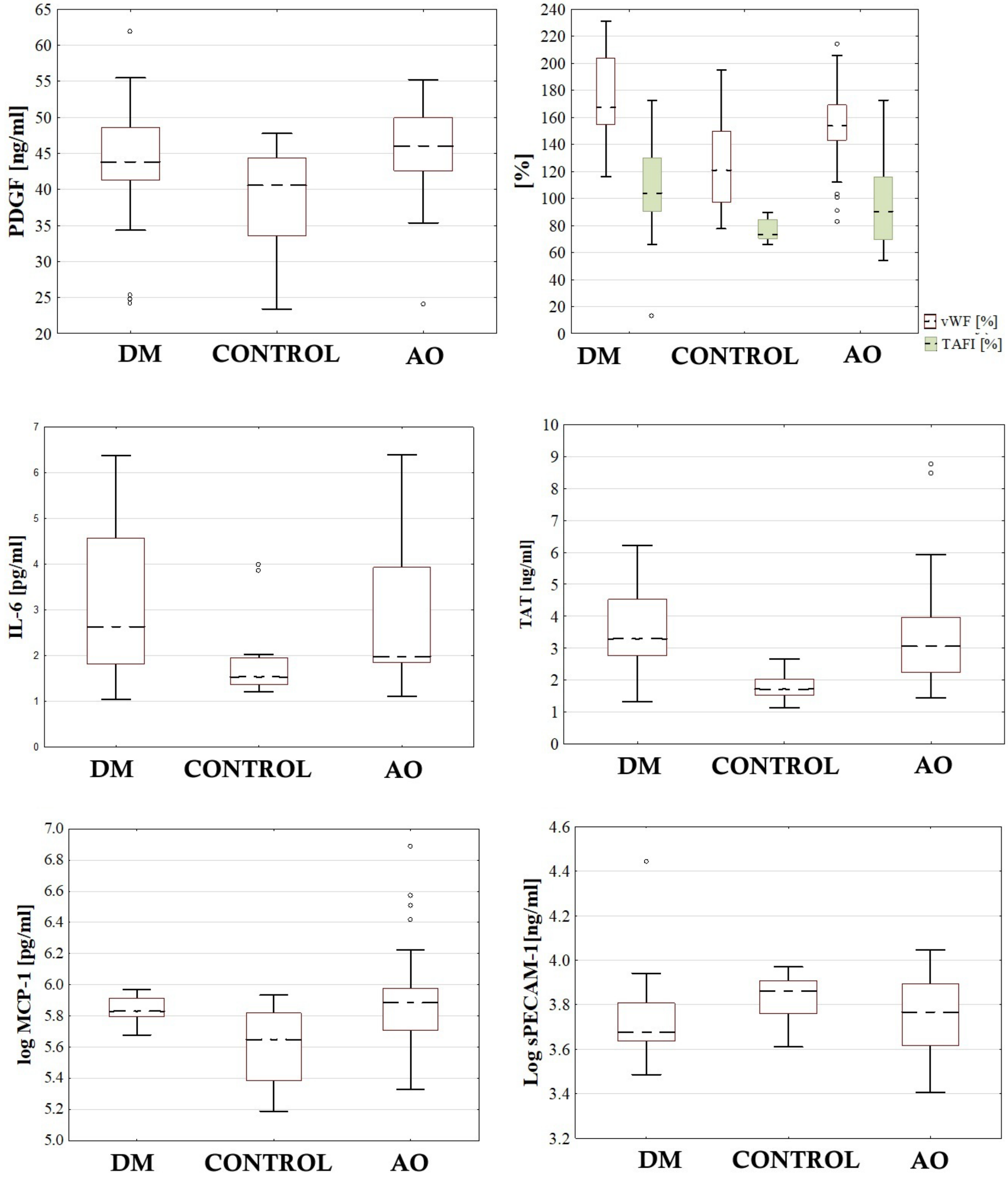

Figure S5. Quantitative assessment of hemostatic and inflammatory factors: concentrations of thrombin-antithrombin III complex (TAT), interleukin 6 (IL-6), percentage of von Willebrand

factor (vWF), percentage of thrombin-activatable fibrinolysis inhibitor (TAFI), concentrations of platelet-derived growth factor (PDGF), monocyte chemoattractant protein 1 (MCP-1), and soluble platelet-endothelial cell adhesion molecule-1 (sPECAM-1) in blood samples from patients with diabetic macroangiopathy (DM), the control group (CONTROL), and patients with atherosclerosis obliterans (AO).

Based on the conducted statistical analysis, the following findings were observed: a significantly lower vWF concentration in the AO group compared to the DM group (153.45±28.12 vs. 174.36±33.24%; p=0.013); no statistically significant differences in the quantitative measurements of other hemostatic factors between the AO and DM groups; a significant increase in vWF (153.45±28.12 vs. 125.89±31.80%; p<0.001), PDGF (22.06±6.5 vs. 16.42±7.44 ng/ml; p=0.002), TAT (3.34±1.54 vs. 1.77±0.44 µg/ml; p<0.001), IL-6 (2.80±1.59 vs. 1.81±0.81 pg/ml; p=0.013), MCP-1 (377.5±152.37 vs. 282.21±68.00 pg/ml; p=0.002), and TAFI (98.62±32.15 vs. 76.59±8.09%; p=0.009) in the AO group compared to the control group; a significant increase in vWF (174.36±33.24 vs. 125.89±31.80%; p<0.001), PDGF (20.91±8.5 vs. 8.64±7.44 ng/ml; p=0.027), TAT (3.62±1.18 vs. 1.77±0.44 µg/ml; p<0.001), IL-6 (3.17±1.53 vs. 1.81±0.81 pg/ml; p=0.001), MCP-1 (344.45±28.70 vs. 282.21±68.00 pg/ml; p=0.035), and TAFI (108.7±33.68 vs. 76.59±8.09%; p<0.001) in the DM group compared to the control group. Statistical analysis considering gender revealed: significantly higher TAT (3.63±1.67 vs. 1.78±0.47 µg/ml; p<0.001), IL-6 (3.21±1.82 vs. 1.92±0.88 pg/ml; p=0.034), MCP-1 (353.69±98.58 vs. 272.39±69.07 pg/ml; p=0.005), and TAFI (108.97±35.43 vs. 76.40±7.48%; p=0.026) in women with AO compared to women in the control group. Significantly higher levels of TAT (3.22±1.50 vs. 1.77±0.35 µg/ml; p=0.020), PDGF (23.13±6.24 vs. 12.75±7.99 ng/ml; p=0.004), and vWF (154.10±26.91 vs. 88.03±8.03%; p<0.001) in men with AO compared to men in the control group; a significantly lower sPECAM-1 concentration (42.98±6.98 vs. 52.51±2.80 ng/ml; p<0.001) in men with AO compared to men in the control group; significantly higher levels of vWF (172.34±29.94 vs. 88.03±8.03%; p<0.001), PDGF (19.87±8.39 vs. 12.75±7.99 ng/ml; p=0.04), TAT (3.59±0.94 vs. 1.77±0.35 µg/ml; p=0.005), IL-6 (3.08±1.38 vs. 1.44±0.35 pg/ml; p=0.034), and TAFI (106.46±26.66 vs. 77.24±11.31%; p=0.041) in men with DM compared to men in the control group; a significantly lower sPECAM-1 concentration (39.91±4.62 vs. 52.51±2.80 ng/ml; p<0.001) in men with DM compared to men in the control group; no significant differences in hemostatic factors between AO and DM in both women and men. Statistical analysis considering gender within diseases revealed: a significantly lower sPECAM-1 concentration (39.91±4.62 vs. 50.16±18.79 ng/ml; p=0.002) in men with DM compared to all DM patients; a significantly higher sPECAM-1 concentration (52.51±2.80 vs. 46.50±4.26 ng/ml; p=0.007) in men from the control group compared to all individuals in the control group, as well as in men from the control group compared to women (52.51±2.80 vs. 48.35±4.02 ng/ml; p=0.048); a significantly higher vWF concentration (136.71±27.21 vs. 88.03±8.03%; p=0.002) in women from the control group compared to men in the control group; a significantly lower vWF concentration (88.03±8.03 vs. 125.89±31.80%; p=0.012) in men from the control group compared to all individuals in the control group; no significant differences in hemostatic factor levels between women, men, and all AO patients.
